# Neuroprotective and Anti-inflammatory Effects of Rubiscolin-6 Analogs with Proline Surrogates in Position 2

**DOI:** 10.1007/s11064-023-04070-z

**Published:** 2023-12-20

**Authors:** Renata Perlikowska, Joana Silva, Celso Alves, Patricia Susano, Małgorzata Zakłos-Szyda, Agnieszka Skibska, Anna Adamska-Bartłomiejczyk, Karol Wtorek, Jean-Claude do Rego, Jean-Luc do Rego, Alicja Kluczyk, Rui Pedrosa

**Affiliations:** 1 Department of Biomolecular Chemistry, Faculty of Medicine, Medical University, Mazowiecka 6/8, 92-215 Lodz, Poland; 2 MARE—Marine and Environmental Sciences Centre, ARNET - Aquatic Research Network, Politécnico de Leiria, 2520-630 Peniche, Portugal; 3 MARE—Marine and Environmental Sciences Centre, ARNET - Aquatic Research Network, ESTM, Politécnico de Leiria, 2520-614 Peniche, Portugal; 4 Institute of Molecular and Industrial Biotechnology, Faculty of Biotechnology and Food Sciences, Lodz University of Technology, Stefanowskiego 2/22, 90-537 Lodz, Poland; 5 Platform of Behavioural Analysis (SCAC), Inserm US51 - CNRS UAR2026 HeRaCLes, Institute For Research and Innovation in Biomedicine (IRIB), University of Rouen Normandy, Rouen, France; 6 Faculty of Chemistry, University of Wroclaw, 50-383 Wroclaw, Poland

**Keywords:** Neuroprotection, Inflammation, Antioxidant activity, 6-hydroxydopamine, Peptides, Natural compounds, Apoptosis, Oxidative stress

## Abstract

Naturally occurring peptides, such as rubiscolins derived from spinach leaves, have been shown to possess some interesting activities. They exerted central effects, such as antinociception, memory consolidation and anxiolytic-like activity. The fact that rubiscolins are potent even when given orally makes them very promising drug candidates. The present work tested whether rubiscolin-6 (R-6, Tyr-Pro-Leu-Asp-Leu-Phe) analogs have neuroprotective and anti-inflammatory effects. These hypotheses were tested in the 6-hydroxydopamine (6-OHDA) injury model of human neuroblastoma SH-SY5Y and lipopolysaccharide (LPS)-stimulated RAW 264.7 macrophages. The determination of reactive oxygen species (ROS), mitochondrial membrane potential (MMP), Caspase-3 activity, lipid peroxidation and nitric oxide (NO) production allowed us to determine the effects of peptides on hallmarks related to Parkinson’s Disease (PD) and inflammation. Additionally, we investigated the impact of R-6 analogs on serine-threonine kinase (also known as protein kinase B, AKT) and mammalian target of rapamycin (mTOR) activation. The treatment with analogs **3** (Tyr-Inp-Leu-Asp-Leu-Phe-OH), **5** (Dmt-Inp-Leu-Asp-Leu-Phe-OH) and **7** (Tyr-Inp-Leu-Asp-Leu-Phe-NH_2_) most effectively prevented neuronal death via attenuation of ROS, mitochondrial dysfunction and Caspase-3 activity. Peptides **5** and **7** significantly increased the protein expression of the phosphorylated-AKT (p-AKT) and phosphorylated-mTOR (p-mTOR). Additionally, selected analogs could also ameliorate LPS-mediated inflammation in macrophages via inhibition of intracellular generation of ROS and NO production. Our findings suggest that R-6 analogs exert protective effects, possibly related to an anti-oxidation mechanism in in vitro model of PD. The data shows that the most potent peptides can inhibit 6-OHDA injury by activating the PI3-K/AKT/mTOR pathway, thus playing a neuroprotective role and may provide a rational and robust approach in the design of new therapeutics or even functional foods.

**Supplementary Information:**

The online version contains supplementary material available at 10.1007/s11064-023-04070-z.

## Introduction

Parkinson’s disease (PD) is a progressive neurodegenerative disorder characterized by a degeneration of dopaminergic neurons in the substantia nigra and misfolded α-synuclein proteins. Biological events related to the cause of cell death in PD, include mitochondrial dysfunction, oxidative stress, dysregulation of calcium homeostasis, impaired turnover of mitochondria, and prion-like behaviour of misfolded proteins [1]. In addition, extensive evidence supports the involvement of inflammation and immune dysfunction in PD onset or progression [2]. The excessive production of ROS contributes to oxidative damage of macromolecules, leading to defects in their physiological function. Consequently, mitochondrial dysfunction, neuroinflammation and neuronal damage events are observed. The cellular and molecular mechanisms and their interaction lead to the progressive loss of dopaminergic neurons in PD, and oxidative stress‑mediated neuron damage [3]. The current treatments for PD conditions have been directed at restoring the dopaminergic system and preventing dopaminergic neuronal cell death; however, they are still unsatisfactory. Indisputably, the advent of L-Dopa and other dopaminergic therapies markedly slowed the disease’s progression. However, the response to pharmacological treatment predictability decreases over the years while several complications develop [4]. The current data suggest that the most effective therapeutic strategy can be based on PD prevention or early treatment. Although the molecular mechanisms for PD development are still little defined, the use of integral therapies and the search for new drug candidates seems crucial and will surely permit a more effective treatment of PD.

The plant-derived linear peptide rubiscolin-6 (R-6, Tyr-Pro-Leu-Asp-Leu-Phe, also known as rubixyl), a naturally occurring compound, was isolated from spinach leaves by the pepsin digestion of D-ribulose-1,5-bisphosphate carboxylase/oxygenase (RuBisCo) [5, 6], together with its shorter family member, rubiscolin-5 (R-5, Tyr-Pro-Leu-Asp-Leu). The protonated Tyr at the N-terminus and Pro as the second residue suggests that rubiscolins might have opioid activity [7]. Soon, rubiscolins were found to display affinity and selectivity towards δ-opioid receptors, and their oral administration exhibited antinociceptive effects in mice [5]. Moreover, R-6 showed promising results on memory consolidation after intracerebroventricular (icv) and oral administration in mice [8]. The intraperitoneal (ip) and oral delivery of R-6 mediated an anxiolytic effect via activating σ_1_ and dopamine D_1_ receptors [9]. The peptide displayed orexigenic activity and stimulated food intake in normal conditions [10, 11]; however, in the case of a high-fat diet, it exhibited anorexigenic activity [12]. Moreover, R-6 showed the enhancement of glucose uptake in skeletal muscle [13] and the antidepressant-like effect in restraint-stressed mice [14]. Cassell et al. [15] partially confirmed the potency and actions of rubiscolins as G-protein-biased δ opioid receptor peptides, while Kurasawa et al. [16] extended their in vitro profiles to reveal that rubiscolins may act as partial agonists for the µ-/δ-opioid receptor heteromers, with limited effects on endogenous ligands or opioid analgesics that activate µ- or κ-opioid receptor.

Additionally, our previously published neuroprotective study showed that R-6 possesses neuroprotective potential in the neuroblastoma cells (SH-SY5Y) damage model caused by 6-hydroxydopamine (6-OHDA) treatment [17]. When SH-SY5Y cells were treated with 6-OHDA (100 µM) in the presence of R-6 (0.1–10 µM) for 24 h, we observed a significant capacity to recover the neurotoxicity induced by neurotoxin treatment. Rubiscolin-6 prevented cell death and significantly reduced the reactive oxygen species (ROS) levels. Additionally, this plant-derived peptide markedly ameliorates the mitochondrial membrane potential (MMP) changes caused by the neurotoxin, and the observed effect was in a range of 50–70% when compared to 6-OHDA alone. Since R-6 showed neuroprotective activity, we decided to evaluate if R-6 analogs can protect neurons against 6-OHDA-induced injury. As mentioned above, neuroprotection can rely on mechanisms involved in ROS limitation and the decrease of inflammatory mediators’ release. It is known that ROS production increased due to inflammation. Many studies link neuroinflammation with the development of neurodegenerative diseases contributing to brain injury [2, 3]. Therefore, the present study also examined the preventive effects of selected peptides on lipopolysaccharide (LPS)-activation of murine macrophages RAW 246.7 cell line.

**Fig.1:**
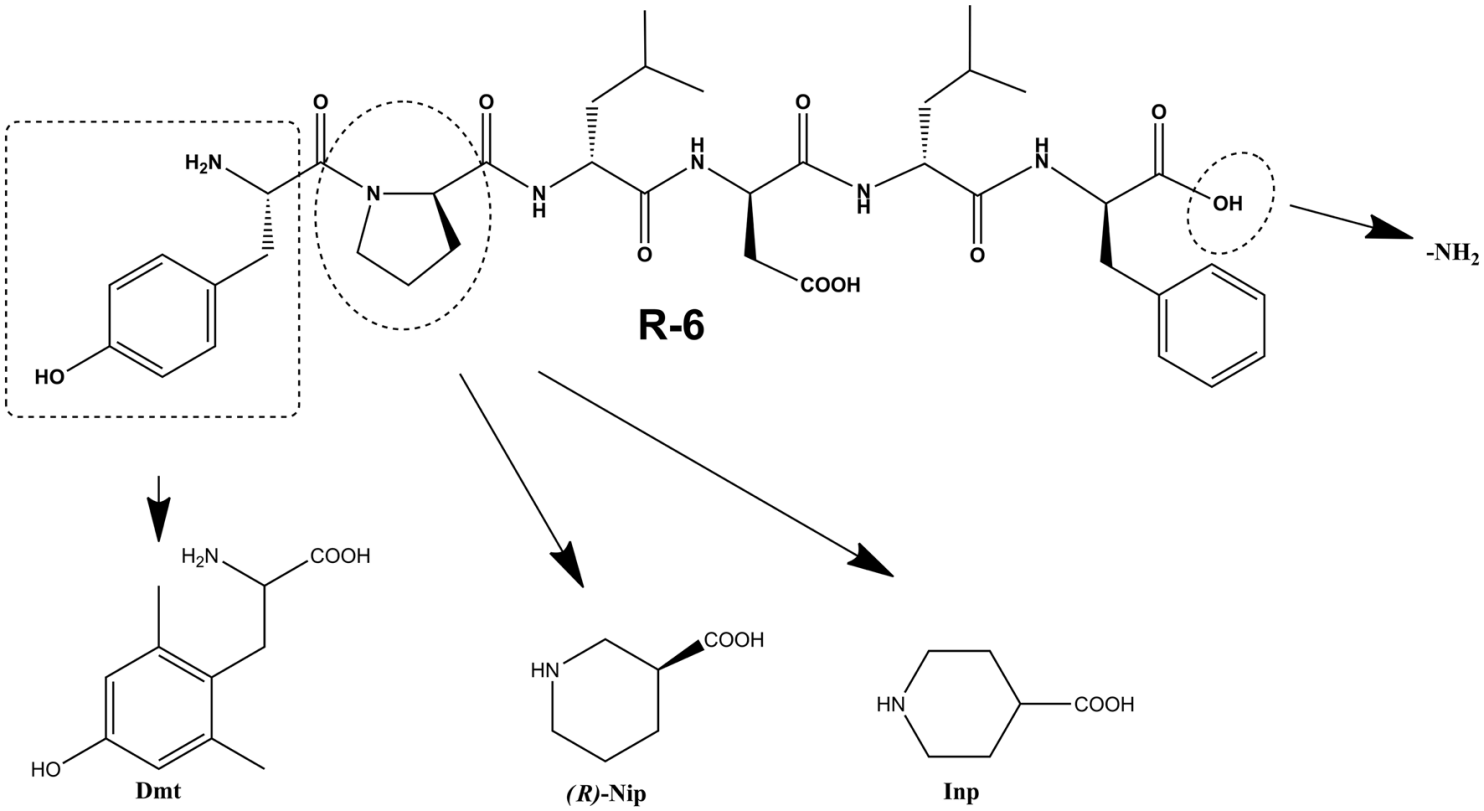
Structure of rubiscolin-6 (R-6) and unnatural amino acids incorporated in positions 1 and 2 (modified positions in the R-6 structure are pointed out with dotted lines)

In this study, the structure of R-6 was modified by incorporating unnatural amino acids. Primarily, we focused on the presence of Pro^2^ residue, a spacer connecting protonated N-terminal Tyr residue in position 1 with an aliphatic amino acid (Leu) in position 3. The occurrence of Pro in the peptide sequence determines important conformational features because this amino acid’s cyclic structure limits the rotation angle around the α-carbon and nitrogen within the peptide bond, consequently introducing a fixed bend into the peptide chain [18, 19]. When most peptide bonds adopt the *trans* conformation, Pro residues exhibit a higher preference to form *cis/trans* isomerization, which is essential in developing the appropriate spatial orientation of adjacent amino acids and influences biological activity. Moreover, the Pro residue confers stability against most proteases and probably makes R-6 active after oral administration [6, 8, 20]. To investigate structural requirements for position 2, R-6 analogs incorporating, instead of Pro, unnatural amino acids with six-membered heterocyclic rings, such as piperidine 3- or 4-carboxylic acids ((*R*)Nip and Inp, respectively) were synthesized. These six-membered rings have a rigid conformation and a preference for substituents (a carboxyl group) to occupy an equatorial position, which may alter the spatial orientation of aromatic pharmacophore residues. Additionally, we diversified the sequences by introducing 2’,6’-dimethyltyrosine (Dmt) instead of Tyr in position 1 and amidating the C-terminus (Fig. 1). Proposed modifications have been widely explored in our earlier opioid peptide studies [21–25]. Introduction of (*R*)-Nip residue in endomorphin structures (Tyr-*(R)*Nip-Trp-Phe-NH_2_ and Tyr-*(R)*Nip-Phe-Phe-NH_2_) turned out to be favourable for improving µ-opioid receptor affinity [21], while Dmt in position 1 led to obtaining analogs (Dmt-*(R)*Nip-Trp-Phe-NH_2_ and Dmt*-(R)-*Nip-Phe-Phe-NH_2_) with exceptional µ-opioid receptor affinity, high stability against enzymatic degradation in rat brain homogenate, and unusual antinociceptive potency [23].

This time, the influence of the R-6 modifications on the receptor binding, functional study and enzymatic stability was reported, as well as the antioxidant, neuroprotective and anti-inflammatory activities. We explored whether the neuroprotective effects of the most potent analogs after 6-OHDA injury are regulated through the phosphatidylinositol 3-kinase/protein kinase B (PI3-K/AKT) and mammalian target of rapamycin (mTOR) signaling pathway. Furthermore, the biological profile of the most promising analogs was initially examined in in vivo mouse locomotor activity assay.

## Materials and Methods

### Peptides Synthesis

Peptides were synthesized manually by standard solid-phase procedures (SPPS) using techniques for 9-fluorenylmethoxycarbonyl (Fmoc)-protected amino acids on Fmoc-Phe Wang (100–200 mesh, 0.69 mM/g, Novabiochem) or MBHA Rink-Amide peptide resin (100–200 mesh, 0.80 mM/g, Novabiochem) and 2-(1H-benzotriazol-1-yl)-1,1,3,3-tetramethyluronium tetrafluoroborate (TBTU) as a coupling agent. The protocol is routinely used in The Department of Biomolecular Chemistry (Medical University of Lodz, Poland) and was described earlier [23]. Crude peptides were purified by semi-preparative reversed-phase HPLC using Waters Breeze instrument on a Vydac C_18_ column (10 μm, 22 × 250 mm), flow rate 2 mL/min, 20 min linear gradient from water/0.1% (v/v) TFA to 80% acetonitrile/20% water/0.1% (v/v) TFA. The analytical HPLC employing a Vydac C_18_ column (5 μm, 4.6 × 250 mm), flow rate 1 mL/min, and the same solvent system over 50 min was used to verify the purity of the final peptides. Final products were obtained with a purity greater than 95%. High-resolution mass spectrometry with electrospray ionization (HR-ESI-MS), performed on a Bruker micrOTOF-Q (time-of-flight) mass spectrometer (peptide R-6) an FTICR (Fourier transform ion cyclotron resonance) Apex-Qe Ultra 7 T mass spectrometer (analogs 1–7, Bruker Daltonics, Bremen, Germany) equipped with standard ESI source, confirmed the mass identity of all synthesized peptides. ^1^H NMR spectra were recorded with a Bruker Avance II Plus spectrometer (Bruker BioSpin, Rheinstetten, Germany) operating at a 1H frequency 700.16 MHz. The instrument was equipped with 5 mm Z-gradient broadband inverse probe (BBI). All experiments were performed at 300 K. The chemical shifts were referenced to the DMSO-d_6_ signal at 2.50 ppm. The selected solvent was considered an excellent mimic of the biological environment for the analysis of opioid peptides. The detailed analytical data of the synthesized peptides are provided in Table 1 and the Supplementary Information (Supplementary data, Table S1, Figs. S2–S16).

**Table 1:** Physicochemical data and degradation half-lives of R-6 and its analogs

No	Sequence	Formula	Molecular weight [MW]	*m/z* [M + H]^+^		Purity (%)^a^	RP-HPLC Rt [min]^b^	T_0.5_ (min)
				calculated	found			
R-6	Tyr-Pro-Leu-Asp-Leu-Phe-OH	C_39_H_54_N_6_O_10_	766.8803	767.3974	767.3993	98	16.38	14.28 ± 0.80
1	**Dmt**-Pro-Leu-Asp-Leu-Phe-OH	C_41_H_58_N_6_O_10_	794.9334	795.4287	795.4260	95	17.05	20.75 ± 0.85***
2	Tyr**-(*R*)-Nip**-Leu-Asp-Leu-Phe-OH	C_40_H_56_N_6_O_10_	780.9068	781.4131	781.4111	98	16.24	25.54 ± 0.40***
3	Tyr-**Inp**-Leu-Asp-Leu-Phe-OH	C_40_H_56_N_6_O_10_	780.9068	781.4131	781.4096	97	16.46	28.60 ± 0.95***
4	**Dmt-(*R*)-Nip**-Leu-Asp-Leu-Phe-OH	C_42_H_60_N_6_O_10_	808.9600	809.4444	809.4424	98	16.78	30.35 ± 1.20***
5	**Dmt-Inp**-Leu-Asp-Leu-Phe-OH	C_42_H_60_N_6_O_10_	808.9600	809.4444	809.4426	98	16.84	38.55 ± 0.70***
6	Tyr-Pro-Leu-Asp-Leu-Phe-**NH**_**2**_	C_39_H_55_N_7_O_9_	765.8955	766.4134	766.4059	95	15.98	23.22 ± 1.05***
7	Tyr-**Inp**-Leu-Asp-Leu-Phe-**NH**_**2**_	C_40_H_57_N_7_O_9_	779.9221	780.4290	780.4192	98	16.16	30.74 ± 0.35***

***p < 0.001 as compared to respective R-6 using one-way ANOVA followed by the Student–Newman–Keuls test

^a^Determined by RP HPLC

^b^RP HPLC performed on a C_18_ column, mobile phase from 100% water/0.1% (v/v) TFA to 80% CH_3_CN/20% water/0.1% (v/v) TFA. All values are expressed as the mean ± standard error of the mean (SEM) of four independent experiments performed in duplicate

### Opioid Receptor Binding Assays

The opioid receptor binding assays were performed using the modified method described elsewhere [23]. Briefly, commercial membranes of Chinese Hamster Ovary (CHO) cells transfected with human opioid receptors were used. The binding affinities for µ-, δ- and κ-opioid receptors were determined by radioligand competition analysis using [^3^H]DAMGO, [^3^H]deltorphin-2, and [^3^H]U-69593, respectively. Membranes were incubated at 25 °C for 120 min with appropriate concentrations of a tested peptide in the presence of 0.5 nM radioligand in a total volume of 0.5 mL of 50 mM Tris/HCl (pH 7.4) containing bovine serum albumin (BSA) (1 mg/mL), bacitracin (50 µg/mL), bestatin (30 µM), and captopril (10 µM). Non-specific binding was determined in the presence of 1 µM naloxone. Incubations were terminated by the rapid filtration through the GF/B Whatman (Brentford, UK) glass fibre strips (pre-soaked for 2 h in 0.5% (v/v) polyethylamine) using Millipore Sampling Manifold (Billerica, MA, USA). The filters were washed thrice with 4 mL of ice-cold Tris buffer solution. Packard Tri-Carb 2100 TR liquid scintillation counter (Ramsey, MN, USA) was used to measure the bound radioactivity after overnight extraction of the filters in 4 mL of a Perkin Elmer Ultima Gold scintillation fluid (Wellesley, MA, USA). The data were analyzed by a nonlinear least square regression analysis computer program Graph Pad PRISM 6.0 (Graph Pad Software Inc., San Diego, CA, USA). The values of the inhibitory constants (K_i_) were calculated according to the equation of Cheng and Prusoff [26].

### Calcium Mobilisation Assay

Chinese Hamster Ovary (CHO) cells stably co-expressing human recombinant µ- or κ-opioid receptors and the C-terminally modified G_αqi5_, and CHO cells co-expressing the human recombinant δ-opioid receptors and the G_αqG66Di5_ chimeric protein (a generous gift from Prof. Girolamo Calo, University of Padova, Italy) were used in calcium mobilization assay, that was performed as reported in detail elsewhere [25]. Cells were cultured in Dulbecco’s Modified Eagle’s Medium (DMEM)/Ham’s F12 (1:1) culture medium supplemented with 10% (v/v) Fetal Bovine Serum (FBS), penicillin (100 IU/mL), streptomycin (100 µg/mL), L-glutamine (2 mM), fungizone (1 µg/mL), geneticin (G418; 200 µg/mL) and hygromycin B (100 µg/mL). Cell cultures were kept at 37 °C in 5% CO_2_/humidified air. When confluence was reached (3–4 days), cells were seeded at a density of 50,000 cells/well into 96-well black, clear-bottom plates. After 24 h incubation, the cells were loaded with a medium supplemented with probenecid (2.5 mM), calcium-sensitive fluorescent dye Fluo-4 AM (3 µM), pluronic acid (0.01%), and HEPES (20 mM) and kept for 30 min at 37 °C. Then, the loading solution was aspirated, and 100 µL/well of assay buffer (HBSS supplemented with 20 mM HEPES, 2.5 mM probenecid, and 500 µM Brilliant Black) was added.

The tested peptides were diluted in the HBSS/HEPES (20 mM) buffer (containing 0.005% BSA) and placed into 96-well plates. The range of concentrations tested was 10 µM–1 × 10^–6^ µM. After placing both plates (cell culture and compound plate) into the FlexStation II (Molecular Device, Union City, CA, USA), the online additions were carried out in a volume of 50 µL/well and the fluorescence changes were measured.

Agonist potencies were given as pEC_50,_ representing a negative logarithm of the molar concentration of an agonist that produces 50% of the maximal possible effect. Concentration–response curves were fitted with the four-parameter logistic, nonlinear regression model:

$${\text{Effect}}\,{ = }\,{\text{baseline}}\,{ + }\,\frac{{{\text{E}}_{{{\text{max}}}} {\text{ - baseline}}}}{{{1}\,{ + }\,{10}^{{\left( {{\text{logEC}}_{{{50}}} {\text{ - X}}} \right)\,{\text{n}}}} }}$$

where X is the agonist concentration and n is the Hill coefficient. Ligand efficacies, expressed as the intrinsic activity (α), were calculated as the E_max_ ratio of the tested compound and the standard agonist.

### Degradation by the Brain Homogenate

The degradation studies were performed on the rat brain homogenate according to the modified method described elsewhere [23]. Briefly, rat brains, after isolation and homogenization in 50 mM Tris–HCl (pH 7.4), were stored at − 80 °C until used. During the test, the aliquots (100 µL, 10 mg protein/mL) were incubated with 100 µL of a peptide (0.5 mM) over 0, 7.5, 15, 22.5, 30, and 60 min at 37 °C in a final volume of 200 µL. The reaction was stopped at the required time by placing the tube on ice and acidifying it with 20 µL of 1 M aqueous HCl solution. Then, the aliquots were centrifuged at 20,000×*g* for 10 min at 4 °C. The obtained supernatants were filtered and analyzed by HPLC on a Vydac C_18_ column (5 µm, 0.46 × 250 mm) using the solvent system of 0.1% trifluoroacetic acid (TFA) in water (A) and 80% acetonitrile in water containing 0.1% TFA (B) and a linear gradient of 0–100% B over 25 min. The rate constants of degradation (k) were obtained as described earlier [23] by a least square linear regression analysis of logarithmic peak areas [ln(*A*/*A*_D_)], where *A*—the amount of peptide remaining, *A*_*D*_—the initial amount of peptide, versus time. Degradation half-lives (*t*_1/2_) were calculated from the rate constants as ln 2/*k*.

### Antioxidant Activity

#### 1,1-Diphenyl-2-picrylhydrazyl (DPPH) Radical Scavenging Activity

Peptides were tested using DPPH technique to estimate their hypothetical antioxidant activity. The assay was performed according to the methodology described by [27]. Briefly, 198 µL of the DPPH solution (0.1 mM) was mixed with 2 µL of sample solution (10 µM). Then, the mixture was incubated in the dark for 30 min at room temperature. Absorbance was measured at 517 nm using a microplate reader (Bioteck, Epoch/2 microplate reader, Winooski, VT, USA). The results were expressed in percentage of control.

#### Oxygen Radical Absorption Capacity Method (ORAC)

The ability of peptides to neutralize the peroxyl radicals was evaluated in ORAC according to the described method [27]. Peptides (20 µL) and fluorescein (120 µL; 70 nM) were placed in a microplate well and pre-incubated for 15 min at 37 °C. Then, 60 µL of 2,2ʹ-azobis (2-amidino-propane) dihydrochloride (AAPH) as a radical generator was added in a final concentration of 12 mM. The fluorescence was read at wavelengths of 458 nm (excitation) and 520 nm (emission) and recorded every minute for 240 min. A blank using phosphate buffer instead of the fluorescein and a calibration curve using Trolox as standard (1–8 µM, final concentration) were also carried on in each assay. Final results were presented in µmol of Trolox equivalents/ µM peptide (µmol TE/ µM peptide).

#### Ferric Reducing Antioxidant Power (FRAP)

FRAP assay adapted to microscale with minor modifications [27] was used to measure the ferric reducing ability of peptides. Acetate buffer (0.3 M, pH 3.6), 2,4,6-Tris(2-pyridyl)-*s*-triazine (TPTZ, 10 mM prepared in 40 mM HCl), and ferric solutions (20 mM) were freshly mixed at a ratio of 10:1:1 and place in a 96-well microplate. Then, 198 µL of FRAP reagent and 2 µL of the peptide sample was mixed and incubated at 37 ºC in the dark for 30 min. The absorbance was measured at 593 nm using a microplate reader (Bioteck, Epoch/2 microplate reader, Winooski, VT, USA). The difference between the absorbance of the test sample and the blank reading was calculated and expressed as µM of FeSO_4_/g of the peptide. FeSO_4_ was used as standard.

### Cell Culture Maintenance

The neuroprotective experiments were performed in an in vitro model of a human neuroblastoma cell line (SH-SY5Y) obtained from the German Collection of Microorganisms and Cell Cultures GmbH (DSMZ) Bank (ACC 209). Cells were cultured in Dulbecco’s Modified Eagle’s Medium: Nutrient Mixture F12 (DMEM:F12) supplemented with 1% antibiotic/antimycotic (Amphotericin B, Penicillin and Streptomycin) (Biowest, Nuaillé, France) and 10% (v/v) Fetal Bovine Serum (FBS) (Biowest, Riverside, MO, USA). The anti-inflammatory experiments were performed in an in vitro model of mouse macrophage-like RAW 264.7 cells, purchased from American Type Culture Collection (ATCC, Manassas, VA, USA). Cells were maintained in DMEM medium (Gibco™, Grand Island, NY) and supplemented with 10% bovine calf serum (BCS) (Gibco™, Grand Island, NY) and 1% penicillin–streptomycin (Sigma-Aldrich, St. Louis, MO, USA). Cell cultures were kept at 37 °C in 5% CO_2_/humidified air.

### Cytotoxic and Cytoprotective Activities of Peptides

The cytotoxic and neuroprotective effects of peptides on SH-SY5Y cells were estimated using the 3-(4,5-dimethyl-2-thiazolyl)-2,5-diphenyl-2H-tetrazolium bromide (MTT) method (VWR, Solon, Ohio, USA) as described earlier [27]. Cells were incubated with peptides (0.1–10 µM) for 24 h to assess their cytotoxic activity. In the neuroprotective model, SH-SY5Y cells were exposed to 6-OHDA (100 µM) in the absence/presence of peptide at non-toxic concentrations (0.1–10 µM). When needed, a 30 min pre-incubation step with β-funaltrexamine (β-FNA, µ-opioid receptor antagonist, 10 µM), naltrindole (NLT, δ-opioid receptor antagonist, 10 µM), 2’-amino-3’-methoxyflavone (PD 98059, MAPK kinase 1 (MEK 1) inhibitor, 50 µM) or 2-(4-morpholinyl)-8-phenyl-4H-1-benzopyran-4-one (LY 294002, PI3-K inhibitor, 20 µM). Stimulation was terminated by aspiration of the medium and adding 100 µL MTT (1.2 mM) per well. Cells were incubated for 1 h at 37 °C. After this time, MTT was removed, and 100 µL DMSO was added. The resulting absorbance was read in a microplate reader (Biotek, Epoch/2 microplate reader, Winooski, VT, USA) at 570 nm.

The cytotoxic and anti-inflammatory effects of peptides on RAW 264.7 cells were estimated using MTT assay. Cells were incubated with peptides (0.1–10 µM) for 24 h to assess their cytoprotective activity. In the anti-inflammatory model, RAW 264.7 cells were exposed to LPS (Millipore Sigma, Darmstadt, Germany) at 1 µg/mL in the absence/presence of peptide at non-toxic concentrations (0.1–10 µM) [28]. Then, the MTT reagent was added to wells, and cells were incubated for 1.5 h at 37 °C. After this time, MTT was removed, 100 µL DMSO was added, and the resulting absorbance was read in a microplate reader (Synergy 2 BioTek Microplate Reader, Winooski, VT, USA) at 570 nm.

Cell metabolic activity (cytotoxicity) was calculated as the percentage of the value obtained for cells incubated with compounds compared to control cells treated with the medium.

### Intracellular ROS Production

The detection of ROS generated in injured cells was evaluated using the 5(6)-carboxy-2ʹ,7ʹ-dichlorofluorescein diacetate (carboxy-H_2_DCFDA) reagent (Invitrogen, Bleiswijk, Netherlands) [27, 29]. After incubation, cells were washed with ice-cold Phosphate-Buffer Saline (PBS). Then, the carboxyl-H_2_DCFDA (100 µL, 20 µM) reagent was added and incubated for 1 h at 37 °C. Finally, the fluorescence intensity was measured at wavelengths of 527 nm (excitation) and 590 nm (emission), and ROS levels were presented in percentage of control (non-treated cells).

### Mitochondrial Membrane Potential (MMP)

Mitochondrial function was assessed by monitoring changes in MMP (**∆Ψm**) using the fluorescent probe, JC-1 fluorescence dye (5,5′,6,6′-tetrachloro-1,1′,3,3′-tetraethyl-imidacarbocyanine iodide, Molecular Probes, T3168, Eugene, Oregon, USA). The assay was performed according to the method described by Silva et al. [27]. Briefly, SH-SY5Y cells were exposed 6 h to 6-OHDA (100 μM) in the presence or absence of peptides. After incubation, cells were washed with ice-cold PBS, and then JC-1 reagent (3 µM, 200 µL) was added for 15 min at 37 °C. Afterwards, JC-1 was removed, and cells were washed with PBS. The formation of JC-1 aggregates (490 nm of excitation and 590 nm of emission) and the monomeric form of JC-1 (490 nm of excitation and 530 nm of emission) was accompanied simultaneously in the plate reader for 30 min. Results were expressed as the ratio of the monomers/aggregates of JC-1 in the percentage of control.

### Caspase-3 Activity

Caspase-3 activity was assessed using “Caspase-3 fluorometric assay” (Sigma, St. Louis, MO, USA) with minor changes adapted by Silva et al. [27]. The cells were treated with 6-OHDA (100 µM) for 6 h in the presence or absence of peptides. After this period, the culture medium was removed, and cells were rinsed with ice-cold PBS and collected by centrifugation at 3300×*g* for 5 min. The pellets were resuspended in lysis buffer, incubated for about 20 min on ice and finally centrifuged at 22,500×*g*, 4 °C, for 20 min. Cell lysates were processed following the manufacturer’s protocol, and fluorescence was read at wavelengths of 360 nm (excitation) and 460 nm (emission). Caspase-3 activity was calculated through the curve slope and presented as Δ fluorescence (a.u.)/mg of protein/min). Staurosporine (STAU, 1 µM) was used as positive control.

### Western Blotting Analysis

The neuroblastoma SH-SY5Y cells were grown in a 6-well plate and when 80–90% confluent treated with 6-OHDA (100 µM) for 24 h. Then cells were stimulated with peptides for 2.5, 5, 7.5 and 10 min (for AKT) or 24 h (for mTOR). When needed, a 30 min pre-incubation step with LY 294002 was included before analogs and 6-OHDA addition. Stimulation was terminated by aspiration of the medium. Cells were washed 2 times with ice-cold PBS, and then 200 µL of ice-cold lysis buffer [150 mM NaCl, 20 mM Tris–HCl, glycerol, 5 mM EDTA, 0.5 mM phenylmethylsulphonylfluoride (PMSF), 10% Triton X-100, 100 mM protease inhibitors, pH 7.4] was added. Cells were incubated on ice for 15 min. Lysates were then centrifuged at 12,000×*g* for 10 min at 4 °C, and the supernatants were used as the total cell lysates. The protein content in the supernatants was quantified using the Bio-Rad protein assay kit. Equal amounts of protein were separated on 10% sodium dodecyl sulfate (SDS)-polyacrylamide gels and transferred to polyvinylidene difluoride membranes from (PVDF, Millipore, Cork, Ireland). Non-specific binding was blocked with tris-buffered saline Tween-20 (TBS-T: 150 mM NaCl, 20 mM Tris–HCl, 0.1% Tween 20, pH 7.5) containing 5% nonfat milk for 2 h at room temperature. Then, the membrane was incubated overnight at 4 °C with one of the following specific primary antibodies: Vinculin (1:1000 dilution; Cell Signalling Technology, Danvers, MA, USA), p-AKT (1:1000 dilution; Cell Signalling Technology, Danvers, MA, USA), AKT (1:1000 dilution; Cell Signalling Technology, Danvers, MA, USA), p-mTOR (1:1000 dilution; Cell Signalling Technology, Danvers, MA, USA), mTOR (1:1000 dilution; Cell Signalling Technology, Danvers, MA, USA). After incubation, membranes were washed six times per 5 min with TBS-T. In the next step, membranes were incubated with HRP-conjugated secondary antibodies (1:1000 dilution; Cell Signalling Technology, Danvers, MA, USA) for 1 h. Following six washes with TBS-T, the protein bands were detected with the Clarity Western ECL reagent from Bio-Rad. The band intensity was quantified by NIH ImageJ densitometric analysis. For quantification, specific phosphorylation was calculated as the ratio of phosphorylated AKT or mTOR signals to the total Akt or mTOR signal detected. Values of unstimulated samples were set to 1.0. The original immunoblots are provided in Supplementary data.

### Lipid Peroxidation

The level of lipid peroxidation was evaluated after RAW 264.7 cells incubation with peptides for 24 h [30]. Then, the cells were loaded with 50 µM diphenyl-1-pyrenylphosphine (DPPP) probe for 15 min, and fluorescence was read at 360 nm excitation and 460 nm emission wavelengths.

### Nitric Oxide (NO) Production

The level of NO production was determined in RAW 264.7 cells. Briefly, after cells attachment, they were treated with LPS (Millipore Sigma, Darmstadt, Germany) at 1 µg/mL for 24 h, and then the peptides were added for an additional 24 h [28]. After cells’ treatment, the medium was collected, and the accumulation of NO metabolite in the cell culture supernatant was measured using Griess reagent (1% sulfanilamide and 0.1% naphthylethylenediamine dihydrochloride; Sigma Aldrich), where 50 µL of the supernatant was mixed with 50 µL of Griess reagent (40 mg/mL) in a 96-well plate. After incubation at room temperature and darkness for 10 min, the absorbance was measured at 540 nm. Cells treated with LPS without samples were used as the positive control of the inflammatory response.

### In vivo experiments

#### Animals

Male Swiss albino CD1 mice (JANVIER LABS, Le Genest-Saint-Isle, France), weighing 20–26 g, were used in the in vivo experiments. The animals were housed in Makrolon cages (L: 40 cm, W: 25 cm, H: 18 cm; 20 mice/cage), with free access to a standard semisynthetic laboratory diet (RM3, SDS, DIETEX-France, Saint-Gratien, France). All animals were kept in a ventilated room, at a temperature of 22 ± 1 °C, under a 12-h light/12-h dark cycle (lights on between 7:00 a.m. and 7:00 p.m.).

All behavioral experiments were carried out between 9:00 a.m. and 6:00 p.m. in testing rooms adjacent to the animal rooms and were conducted by authorized investigators. Mice were tested only once and sacrificed immediately. Animal manipulations were approved by CEEAN-France (Regional Ethical Committee for Animal Experimentation) and performed according to the European Communities Council Directive (86/609/EEC + 2010/63/UE).

#### Intracerebroventricular Injections

Intracerebroventricular (icv) injections (10 µL/mouse) of R-6 and its analogs were performed in the left brain ventricle of manually immobilized mice, with a Hamilton microsyringe (50 mL, Hamilton, Bonaduz, Switzerland) connected to a needle (length: 3.5 mm, diameter: 0.5 mm), as described elsewhere [31]. Intracerebroventricular injections were performed by an experienced investigator, who frequently controlled the regularity and success of the injections using a methylene blue dye and observed (after sacrifice and frontal brain sectioning) that the injection was successful in more than 95% of trials. It was also verified in several animals, after sacrifice, that the mark of the needle puncture on the parietal bone was located at least 1.5 mm behind the bregma and at least 2.5 mm before the lambda, with laterality between 1 and 2 mm relative to the brain median line. This corresponds to stereotaxic coordinates of the left lateral ventricle in Lehmann’s atlas for mice (Lehmann, 1974): anteriority 2.95e4.15 mm, laterality 0.95e2 mm, and depth 3e4.5 mm. All drugs for icv administration were dissolved in 0.9% NaCl just before treatment. The icv injection method was approved by the Regional Ethical Committee for Animal Experimentation (Normandy; no. N/10-04-04-12).

Tested peptides were dissolved in DMSO and diluted with saline solution to the desired concentrations. Control experiments showed that DMSO had no effect.

#### Locomotor Activity

Locomotor activity was assessed automatically in a computerized actimeter (Omnitech Electronics, Inc., Columbus, Ohio, USA) which monitored horizontal displacements and vertical movements [32]. Ten minutes after the icv injection, mice were placed individually in 20 × 20 × 30 cm compartments in a dimly illuminated and quiet room, and then motor activity was recorded. The responses were expressed as the number of crossed beams by the mouse during three consecutive 20 min periods.

### Data and Statistical Analysis

At least three independent experiments were carried out in triplicate, and results were presented as mean ± standard error of the mean (SEM) and the half-maximal inhibitory concentration (EC_50_). ANOVA with Dunnett’s multiple comparisons of group means analysis was accomplished, and the Tukey’s test was applied for multiple comparisons. In animal studies, differences between groups were assessed by one-way analysis of variance (ANOVA), followed by a post-hoc multiple comparison Student–Newman–Keuls test. Differences were considered significant at the level of 0.05 (*p* < 0.05). GraphPad v8.0 (GraphPad Software, La Jolla, CA, USA) software was used to accomplish the analysis.

## Results

### Peptide Synthesis

Peptides were synthesized by standard solid-phase procedures using techniques for 9-fluorenylmethoxycarbonyl (Fmoc)-protected amino acids on Fmoc-Phe Wang (100–200 mesh, 0.69 mM/g, Novabiochem) or MBHA Rink-Amide peptide resin (100–200 mesh, 0.80 mM/g, Novabiochem). The final purity of all peptides was > 95%. Calculated values for protonated molecular ions agreed with those determined by HR-ESI–MS. The physicochemical data of tested peptides are presented in Table 1, and the detailed analytical data are collected in the Supplementary data (Table S1, Figs. S2–S18).

### Receptor Binding and Functional Activity

The ability of R-6 and its new analogs to bind to µ-, δ- and κ-opioid receptors was determined by competitive binding assays against [^3^H]DAMGO, [^3^H][Ile^5,6^]deltorphin-2 and [^3^H]U-69593, respectively, using membranes of CHO cells transfected with opioid receptors and are summarized in Table 2.

**Table 2:** Receptor binding affinities (K_i_) of R-6 and its analogs at µ-, δ- and κ-opioid receptor

No	K_i_^a^ [nM]		
	MOR	DOR	KOR
R-6	> 1000	11.47 ± 2.25	> 1000
1	598.55 ± 84.49	91.72 ± 29.69	> 1000
2	> 1000	> 1000	> 1000
3	> 1000	734.21 ± 31.27	> 1000
4	> 1000	> 1000	> 1000
5	712 ± 100.51	13.13 ± 3.08	> 1000
6	> 1000	> 1000	> 1000
7	> 1000	773.33 ± 136.88	> 1000

^a^Binding affinities were determined by competitive displacement of the selective radioligands, [^3^H]DAMGO (µ-opioid receptor), [^3^H][Ile^5,6^]deltorphin-2 (δ-opioid receptor), and [^3^H]U-69593 (κ-opioid receptor) using commercial membranes of CHO cells transfected with human opioid receptors. All values are expressed as the mean ± SEM of four independent experiments performed in duplicate

The parent peptide, R-6 bounded only to the δ-opioid receptor, which agreed with earlier results published by Yang et al. [5] and Cassell et al. [15]. Analog **1,** with Dmt in position 1, displayed rather a weak affinity for both receptors; however, the ability to bind to the δ-opioid receptor was stronger. In the case of analogs **2** and **3**, possessed (*R*)Nip and Inp in position 2, respectively, we observed slight differences. The more favorable modification concerned the presence of Inp, thus, peptide **3** bounded slightly to the δ-opioid receptor. Similar observations are made for analogs **4** and **5**, additionally modified in position 1, by introducing Dmt instead of Tyr. Peptide **5**, displayed weak affinity to the µ-opioid receptor and produced enhancement of the δ-opioid receptor affinity. C-terminal amidation, which removes the charge from the C-terminus of a peptide, produced analogs that did not bind to any of the three opioid receptors except analog **7**. In this case, we observed a slight increase in δ-opioid receptor affinity. According to our observations, the stereochemistry of the Pro surrogate played a critical role in the affinities of analogs. Although none of these peptides showed great ability to activate opioid receptors, it is possible to observe that the presence of Inp in position 2 (analogs **3**, **5** and **7**) results in a slight increase of δ-opioid receptor affinity. Additionally, Dmt instead of Tyr in position 1 further enhanced µ-opioid receptor affinity and a slight decrease in selectivity.

**Fig. 2:**
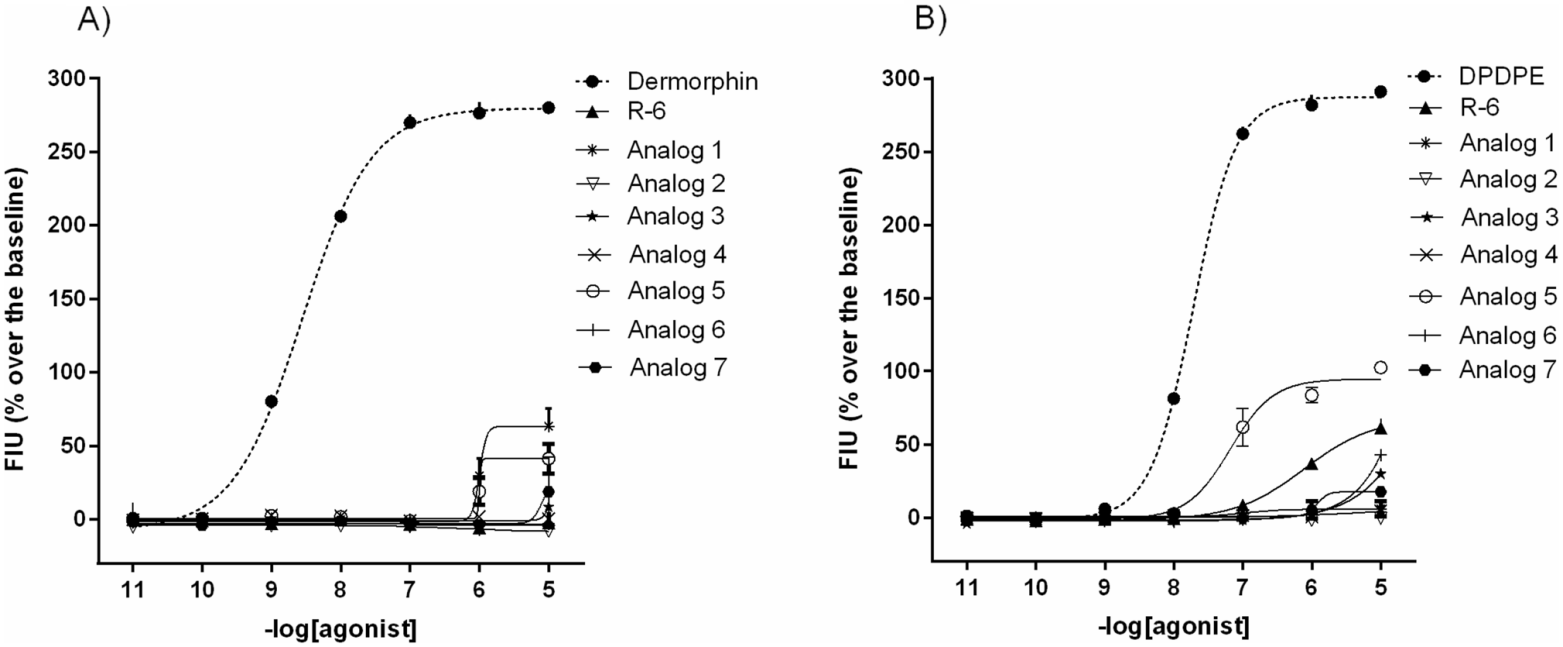
Concentration–response curves for R-6, its analogs, Dermorphin (**A**), and DPDPE (**B**) in calcium mobilization experiments performer in **A** CHO cells co-expressing µ-opioid receptors, **B** CHO cells co-expressing δ- opioid receptors, respectively

The pharmacological profiles of R-6 and its analogs were characterized in calcium mobilization functional assay performed in CHO cells co-expressing µ- or δ- opioid receptors. The concentration–response curves of all tested peptides are shown in Fig. 2A–B, and the calculated agonist potencies (pEC_50_) and efficacies (α) of the analogs are summarized in Table 3. As the reference full agonists for calculating intrinsic activity at the µ- and δ- opioid receptors, Dermorphin and DPDPE were used, respectively. In the CHO cells expressing µ- opioid receptors, only compounds **1** and **5** displayed lower potency (5.70 ± 0.23 and 5.12 ± 0.33, respectively) and efficacy when we compared to 8.26 ± 0.17 for Dermorphin. The remaining peptides were inactive. A similar situation was observed in the case of CHO cells expressing δ- opioid receptors; peptides didn’t mimic the stimulatory effect of DPDPE (7.18 ± 0.26). Among the inactive peptides, only R-6 and analog **5** exhibited a slight potency (5.25 ± 0.45 and 4.65 ± 0.78) and reduced efficacy.

**Table 3:** Effects of R-6 analogs at human recombinant opioid receptors coupled with calcium signaling via chimeric G proteins

Compound	µ-opioid receptors		δ-opioid receptors	
	pEC_50_ (CL_95%_)^a^	α ± SEM	pEC_50_ (CL_95%_)	α ± SEM
Dermorphin	8.26 ± 0.17	1.00	Inactive^a^	
DPDPE	Inactive		7.18 ± 0.26	1.00
R-6	Inactive		5.25 ± 0.45	0.28 + 0.04
1	5.70 ± 0.23	0.22 ± 0.05	Inactive	
2	Inactive		Inactive	
3	Inactive		Inactive	
4	Inactive		Inactive	
5	5.12 ± 0.33	0.11 ± 0.04	4.65 ± 0.78	0.39 ± 0.02
6	Inactive		Inactive	
7	Inactive		Inactive	

Dermorphin and DPDPE were used as reference agonists for calculating intrinsic activity at the µ- and δ-opioid receptors, respectively

^a^Agonist potency values (pEC_50_)

^b^Efficacy values (α); Inactive means that the compound was inactive as an agonist up to 1 µM. All values are expressed as the mean ± SEM of three independent experiments performed in triplicate

### Enzymatic Degradation

The stability of R-6 and its new analogs towards enzymatic degradation was verified by measuring their hydrolysis rates in the presence of rat brain homogenate. Table 1 summarises the half-lives (t_1/2_) determined for tested peptides. All new analogs showed increased stability over R-6. Analogs modified in position 2, were about 2 times more stable than the parent compounds, while additional modification with Dmt seemed to enhance the stability of the analogs further. C-terminal amidation also increased the resistance to enzymatic degradation.

### Antioxidant Activity of Peptides

**Fig. 3:**
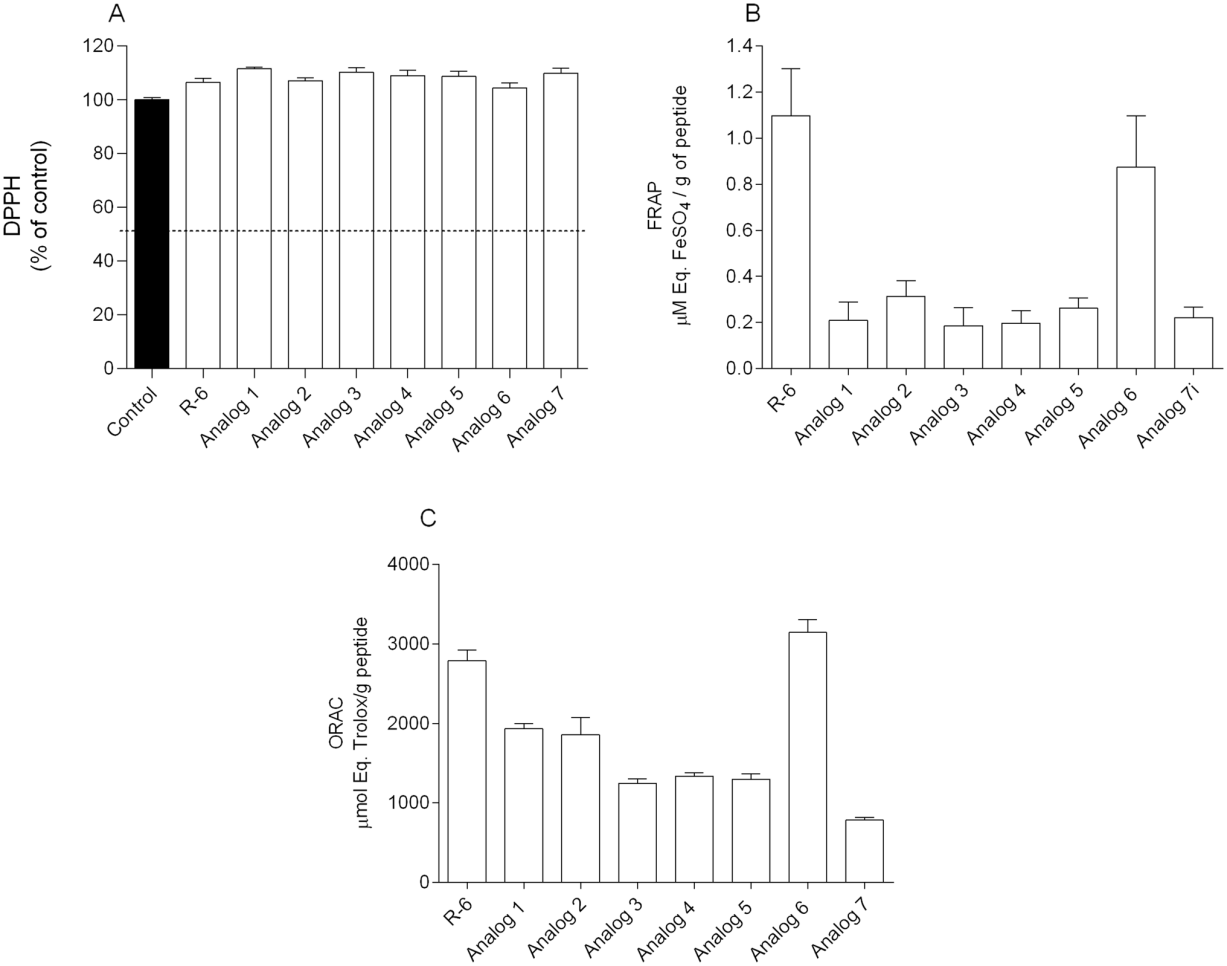
Antioxidant activity of the peptides (10 µM) was evaluated through the DPPH (**A**), FRAP (**B**) and ORAC (**C**) methods. The values in each column represent the mean ± SEM of three or four independent experiments

The antioxidant activity of peptides was evaluated by means of different chemical methods, namely DPPH radical scavenging, FRAP and ORAC. Three independent experiments of each method were carried out with n = 3, and the results are shown in Fig. 3A–C.

**Fig. 4:**
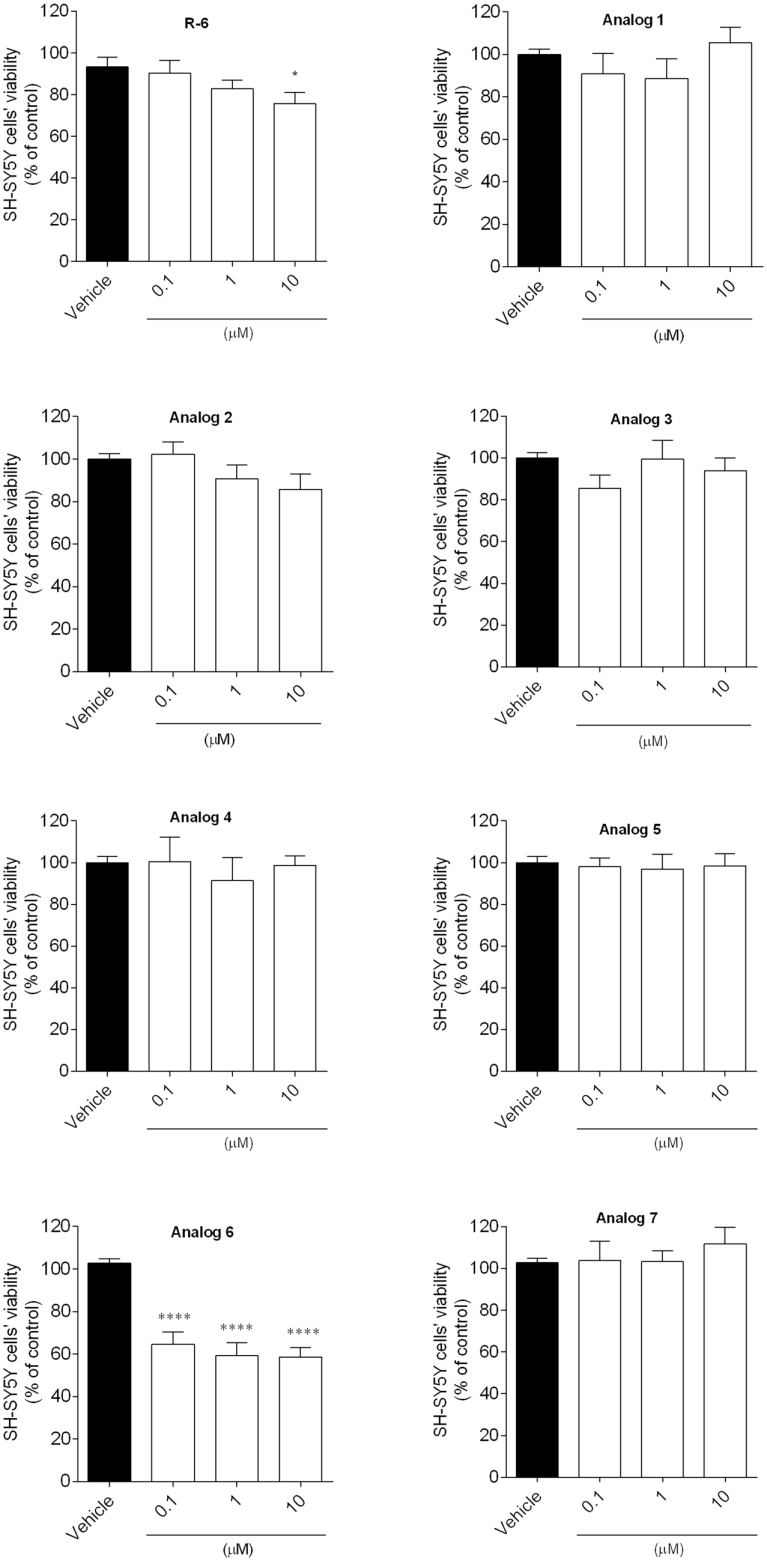
SH-SY5Y cells’ viability when exposed 24 h to peptides (0.1–10 µM). The values in each column represent the mean ± SEM of three or four independent experiments. Symbols represent significant differences (ANOVA, Dunnett’s test), *p < 0.05, ****p < 0.0001 compared to vehicle

**Fig. 5:**
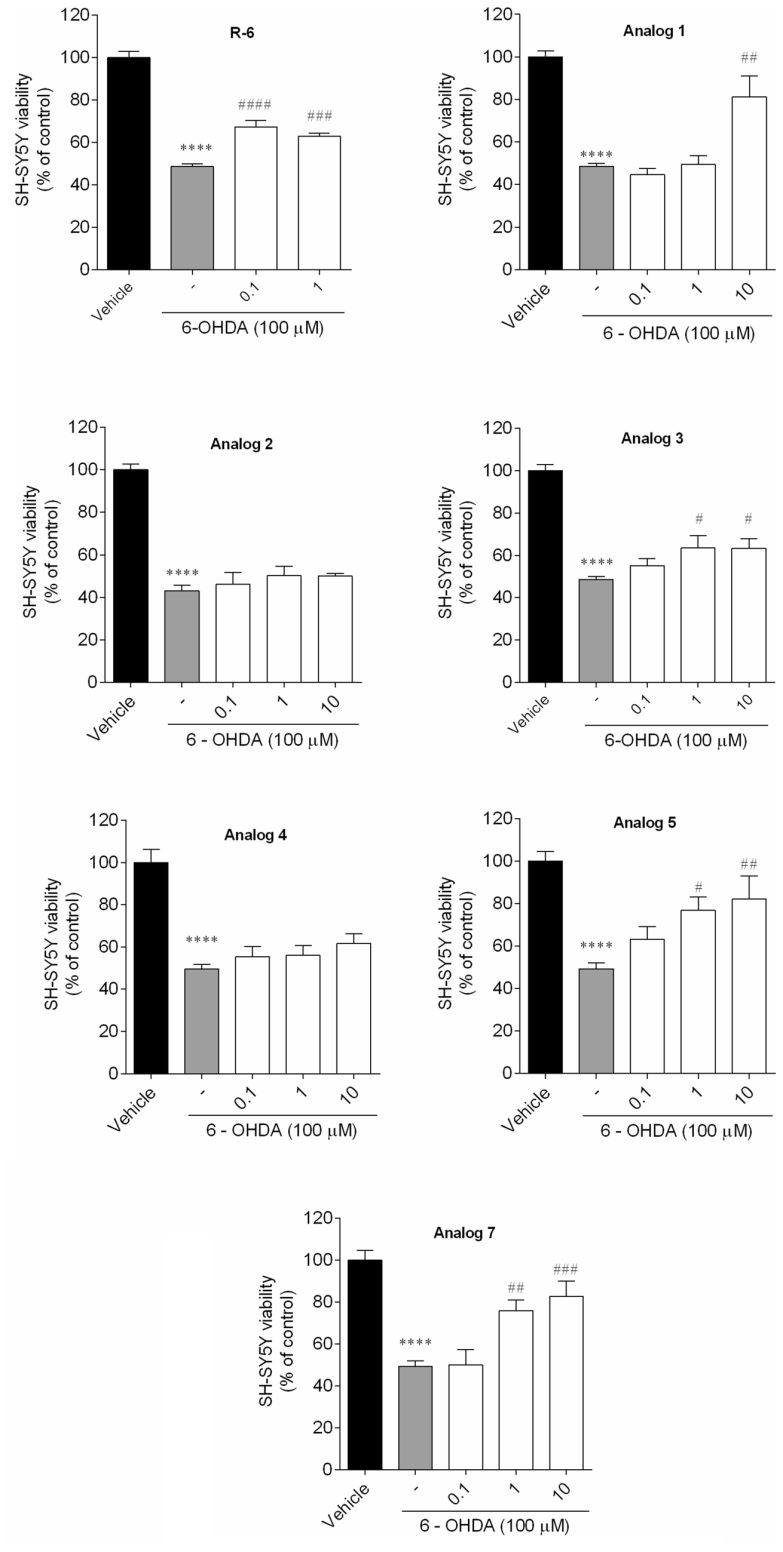
Neuroprotective effects on SH-SY5Y cells treated with 6-OHDA (100 µM) in the presence/absence of peptides (0.1–10 µM, 24 h). The values in each column represent the mean ± SEM of three or four independent experiments. Symbols represent significant differences (ANOVA, Dunnett’s test), ****p < 0.0001 when compared to vehicle and ^#^*p* < 0.05, ^##^*p* < 0.01, ^###^*p* < 0.001 compared to 6-OHDA. (−) 6-OHDA

**Fig. 6:**
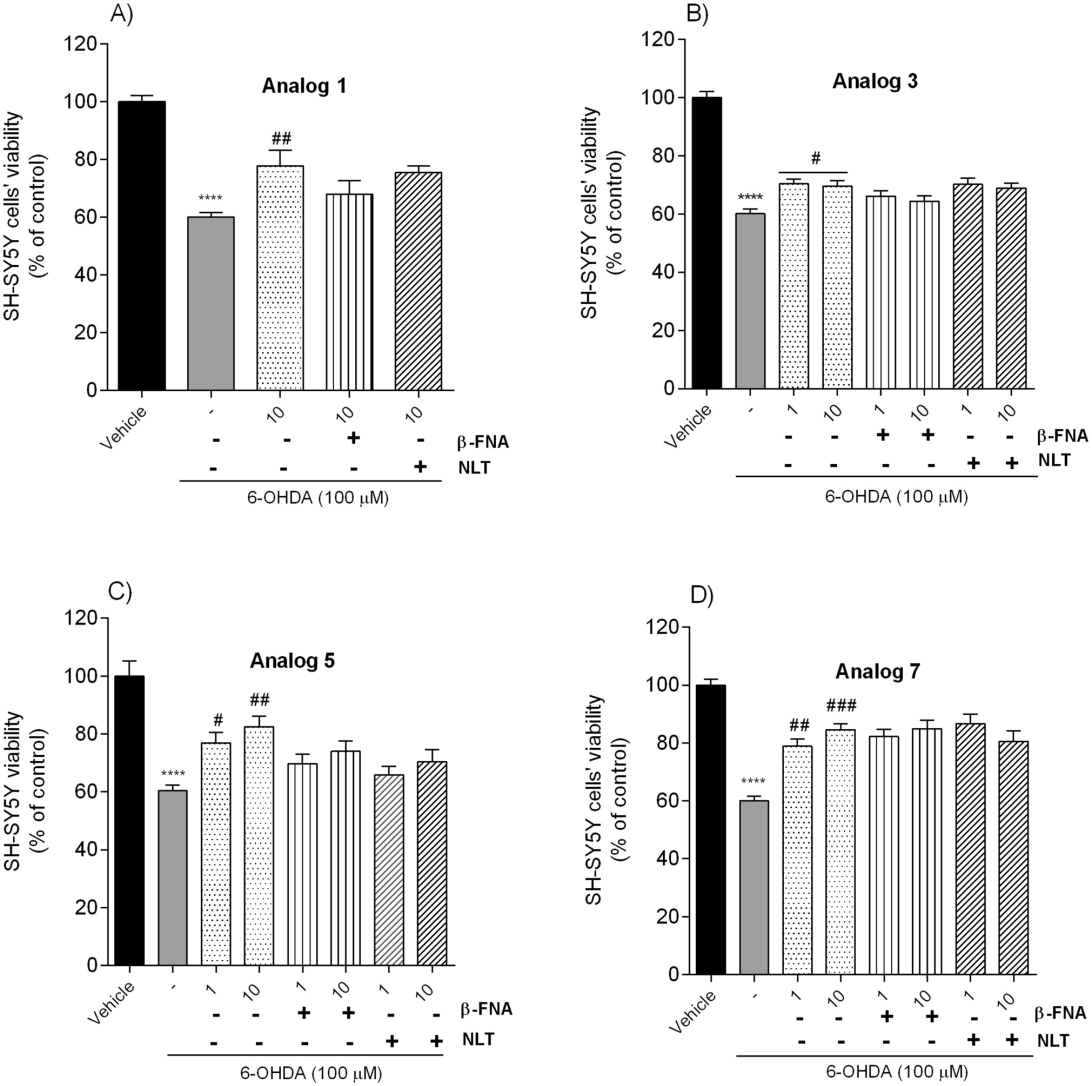
Neuroprotective effect on SH-SY5Y cells treated with 6-OHDA (100 µM) in the presence of peptides: **A** analog **1** (at the dose of 10 µM, 24 h), **B** analog **3**, **C** analog **5**, **D** analog **7** (at the dose of 1 and 10 µM, 24 h), respectively, and β-funaltrexamine (β-FNA, µ-opioid receptor antagonist, 10 µM), or naltrindole (NLT, δ-opioid receptor antagonist, 10 µM). The values in each column represent the mean ± SEM of 3 independent experiments. Symbols represent significant differences (one–way ANOVA, Tukey test) ****p < 0.0001, as compared to a vehicle using; ^#^*p* < 0.05, ^##^*p* < 0.01, ^###^*p* < 0.001 as compared to 6-OHDA; (−) 6-OHDA

None of the peptides could reduce the DPPH radical (Fig. 3A). In FRAP assay (Fig. 3B), it is possible to observe that R-6 (1.09 ± 0.20 µM Eq. FeSO_4_/g of peptide) and its amidated derivative, analog **6**, exhibited the highest ability (0.87 ± 0.22 µM Eq. FeSO_4_/g of peptide) to reduce iron ions when compared to the other peptides. Similar results were observed for the ORAC assay (Fig. 3C), R-6 (2789 ± 131.6 µmol Eq. Trolox/g of peptide), and analog **6** (3143 ± 164.4 µmol Eq. Trolox/g of peptide) displayed the highest ability to neutralize the peroxyl radicals.

### Neuroprotective Activity of Peptides on SH-SY5Y Cells

#### Cytotoxicity of Peptides

The cytotoxic effect of peptides on SH-SY5Y cells’ viability was evaluated, and the obtained data are presented in Fig. 4. The viability of SH‐SY5Y cells treated with different concentrations of peptides (0.1, 1, 10 µM) was determined by MTT assay. Cells were incubated for 24 h with synthesized peptides, and the cell viability was described as the percentage of the surviving control cells. The results showed that analog** 6** significantly decreased the SH-SY5Y cell’s viability when tested at 10 µM, and this concentration has not been further investigated. Thus, the concentrations of peptides that did not induce cytotoxicity were selected to be tested in the neuroprotective assay.

#### Neuroprotective Effects of Peptides on SH-SY5Y Cells

To evaluate the neuroprotective influence of R-6 analogs, the 6-OHDA (100 μM)-treated cells were treated with peptides at 0.1, 1, and 10 µM for 24 h. The effects on cell viability were described as the percentage of the surviving control cells (Fig. 5).

The exposure of SH-SY5Y cells to 6-OHDA (100 µM) for 24 h led to a reduction of cell viability of about 52% (48.63 ± 1.25% of viable cells) when compared to vehicle (100.00 ± 4.60% of viable cells). However, when SH-SY5Y cells were treated with 6-OHDA in the presence of peptides (0.1 − 10 µM), R-6 and its four analogs (**1**, **3**, **5** and **7**) exhibited the capacity to recover the cell death induced by neurotoxin treatment. Parent peptide increased the cell viability at 20% (at the lowest dose) [17], while analog **1** at 30% (at the dose of 10 µM), **3** at 15% (at the dose of 1 and 10 µM, respectively), **5** in 14 − 27%, **7** in 22 and 27% (at the dose of 1 and 10 µM, respectively).

The receptor binding study and calcium mobilization functional assay showed that all studied peptides displayed relatively weak affinity and agonist potency for opioid receptors. As mentioned, only analogs **1**, **3**, **5** and **7** could bind slightly to µ- or δ-opioid receptors. Thus, we decided to determine whether opioid receptors might be involved in the neuroprotective effect of those analogs. To study their possible involvement, SH-SY5Y cells were treated with β-FNA (µ-opioid receptor antagonist, 10 µM) or NLT (δ-opioid receptor antagonist, 10 µM) 30 min before peptides and 6-OHDA (100 µM) addition. Then, according to the described method, after 24 h of incubation, the cell viability was assessed after treatment by the MTT assay. Our observations suggest that the opioid receptors weren’t involved in neuroprotective effects. Concurrent exposure of cells to analogs and opioid receptor antagonists did not abolish the peptide neuroprotection (Fig. 6). However, in the case of analog **5** some slight µ- and δ-opioid receptor antagonist effect is evident. Based on this observation, we can assume that a different signaling cascade may be responsible for the neuroprotective effects of tested peptides.

**Fig. 7:**
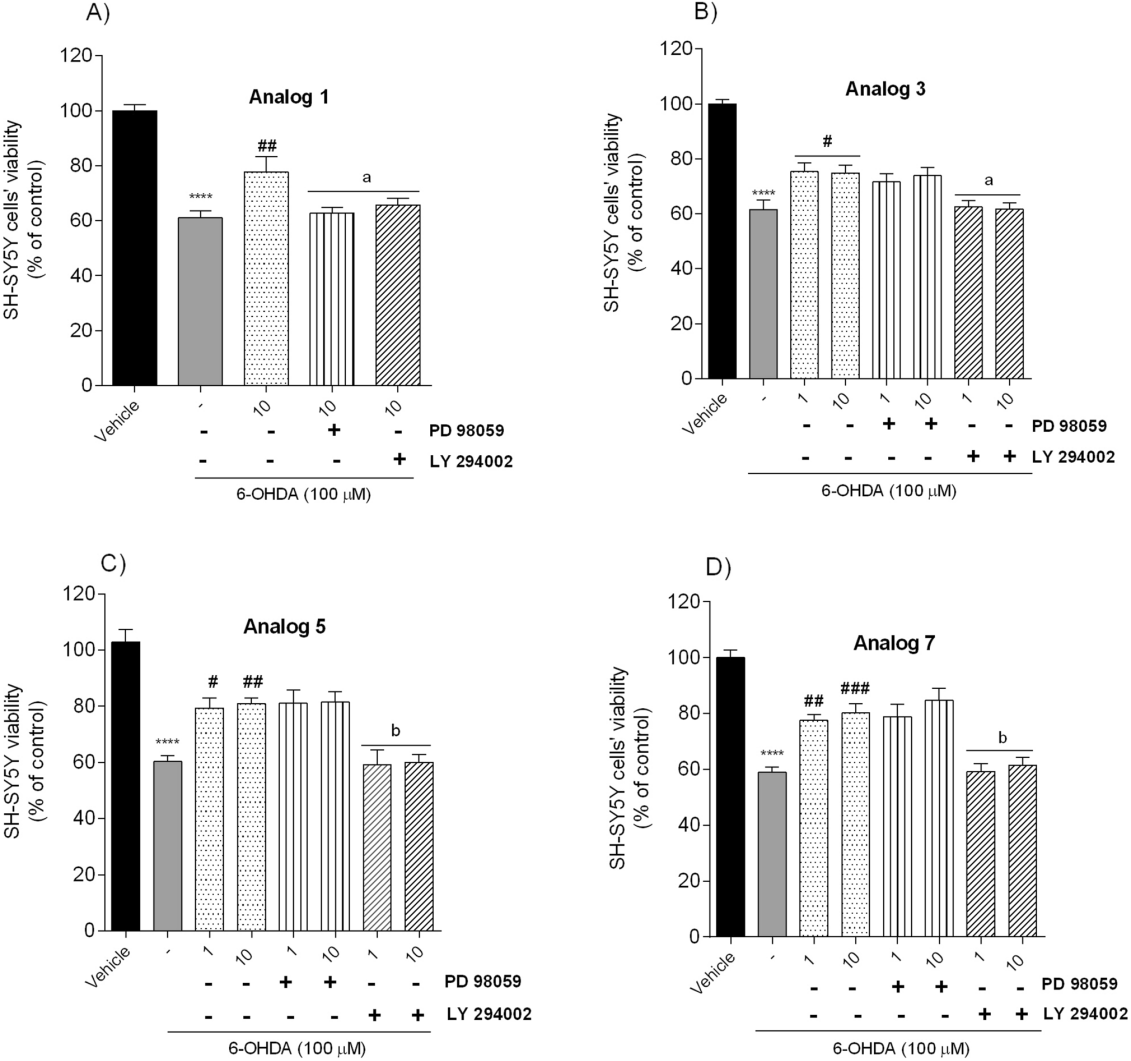
Neuroprotective effect on SH-SY5Y cells treated with 6-OHDA (100 µM) in the presence of peptides **A** analog **1** (at the dose of 10 µM, 24 h), **B** analog **3**, **C** analog **5**, **D** analog **7** (at the dose of 1 and 10 µM, 24 h), respectively, and PD 98059 (50 µM), or LY 294002 (20 µM). The values in each column represent the mean ± SEM of 3 independent experiments. Symbols represent significant differences (one–way ANOVA, Tukey test) ****p < 0.0001, as compared to the vehicle; ^#^*p* < 0.05, ^##^*p* < 0.01, ^###^*p* < 0.001 as compared to 6-OHDA; a *p* < 0.05, b *p* < 0.01 as compared to analog alone, at the dose of 1 and 10 µM, respectively; (−) 6-OHDA

Therefore, we further explored the potential neuroprotective mechanism used by the most potent peptides and incubated SH-SY5Y cells with PD 98059 (MEK 1 inhibitor, 50 µM) or LY 294002 (PI3-K inhibitor, 20 µM) 30 min before peptides and 6-OHDA (100 µM) addition. The effect was examined via MTT assay. Phosphatidylinositol 3-kinase/protein kinase B (PI3-K/AKT) and mitogen-activated protein kinase (MAPK) pathways are crucial for maintaining many physiological functions. They can be a critical link in targeting the modulation of apoptosis, cellular metabolism, cell growth and survival [33]. Trophic factors or G protein-coupled receptor (GPCRs) agonists can activate both signal transduction pathways [34], increasing cell survival. To study the possible involvement of PI3-K/AKT and MAPK in the neuroprotective effect observed for analogs **1**, **3**, **5** and **7**, we examined the impact of PI3-K and MAPK inhibitors. Pre-treatment of cells (30 min) with PD 98059 (50 µM) before 6-OHDA addition didn’t inhibit the neuroprotective effect of tested peptides, while pre-treatment with LY 294002 (20 µM) significantly weakened the neuroprotection induced by analogs **1**, **3**, 5 and **7** (Fig. 7). These observations suggested that PI3-K is involved in the protective effect of peptides abolishing the toxic impact of 6-OHDA.

#### Measurement of Intracellular ROS Formation

**Fig. 8:**
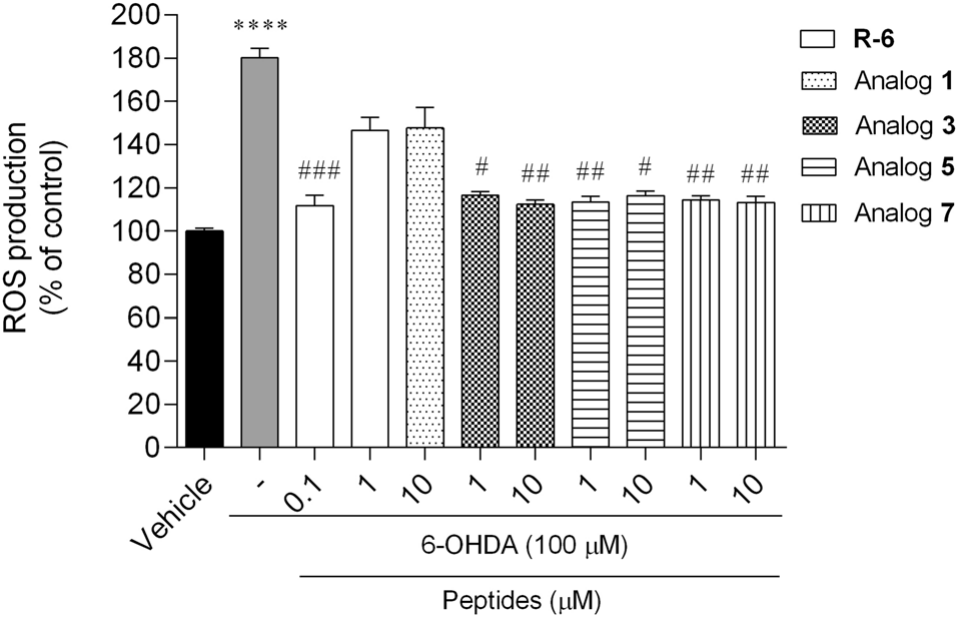
Reactive oxygen species production of SH-SY5Y cells treated with 6-OHDA in the presence/absence of peptides (0.1–10 µM, 6 h). The values in each column represent the mean ± SEM of three or four independent experiments. Symbols represent significant differences (ANOVA, Dunnett’s test), ****p < 0.0001 as compared to vehicle and ^#^*p* < 0.05, ^##^*p* < 0.01, ^###^*p* < 0.001 as compared to 6-OHDA. (−) 6-OHDA

Production of ROS, as one of the crucial hallmarks related to neuronal cell death in PD, was evaluated to understand if the neuroprotective effect of peptides was mediated by its inhibition. The exposure of cells to 6-OHDA (100 µM) led to a marked increase of ROS levels by around 80% compared with vehicle (Fig. 8). The protective effect of R-6 was observed only at the dose of 0.1 µM, where intracellular ROS levels were significantly dumped by about 70%. The analogs **3**, **5** and **7** at both concentrations, 1 and 10 µM, also significantly decreased by about 60% the ROS production stimulated by 6-OHDA treatment.

#### Determination of MMP in SH-SY5Y Cells

**Fig. 9:**
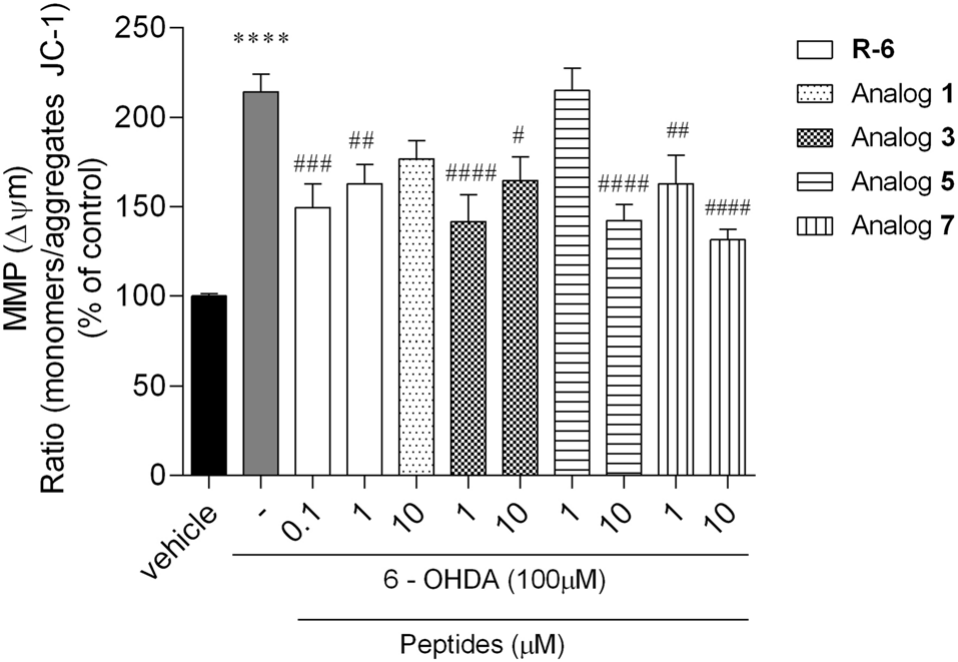
Changes in mitochondrial membrane potential (MMP) of SH-SY5Y cells following treatment with 6-OHDA (100 µM) in the presence/absence of peptides (0.1–10 µM, 6 h). The values in each column represent the mean ± SEM of three or four independent experiments. Symbols represent significant differences (ANOVA, Dunnett’s test), ****p < 0.0001 as compared to vehicle and ^#^*p* < 0.05, ^##^*p* < 0.01, ^###^*p* < 0.001, ^####^*p* < 0.0001 as compared to 6-OHDA. (−) 6-OHDA

Mitochondrial function is a key indicator of cell health and can be assessed by monitoring changes in mitochondrial membrane potential (MMP). The exposure of SH-SY5Y cells to 6-OHDA (100 µM) increased the MMP depolarization (+ 114%) when compared to the vehicle (Fig. 9). Rubiscolin-6 markedly decreased the changes caused by the neurotoxin. In the presence of other peptides (except for analog **1** at 10 µM and analog **5** at 1 µM, respectively), a significant decline in MMP depolarization by about 40 − 80% was observed.

#### Measurement of Caspase-3 Activity

**Fig. 10:**
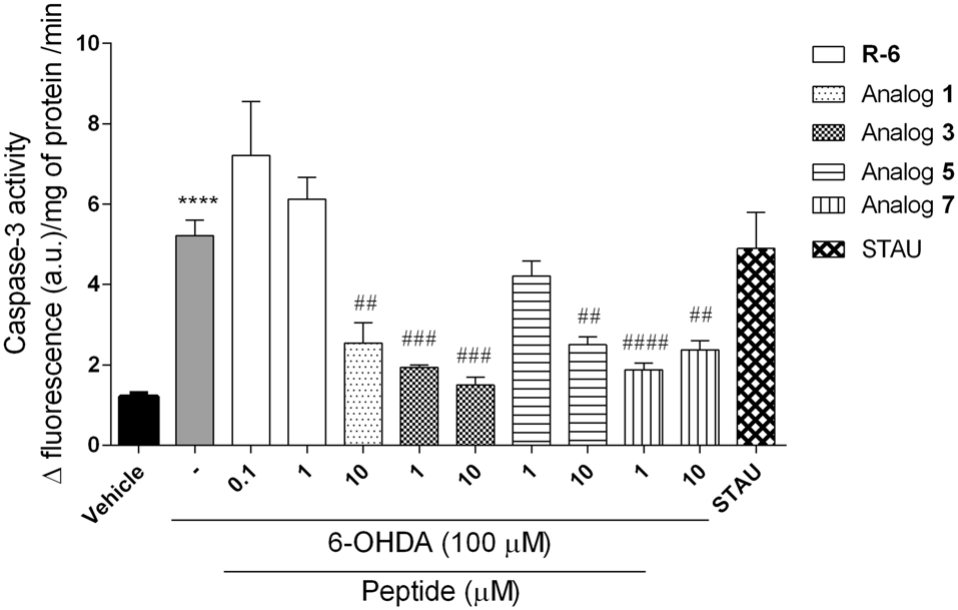
Caspase-3 activity of SH-SY5Y cells treated with 6-OHDA in the presence/absence of peptides (0.1–10 µM, 6 h) and staurosporine (STAU, 1 µM). The values in each column represent the mean ± SEM of three or four independent experiments. Symbols represent significant differences (ANOVA, Dunnett’s test), ****p < 0.0001 as compared to vehicle and ^##^*p* < 0.01, ^###^*p* < 0.001, ^####^*p* < 0.0001 as compared to 6-OHDA. (−) 6-OHDA

The exposure of cells to 6-OHDA (100 µM) and STAU led to a marked increase of Caspase-3 activity (5.22 ± 0.38 Δ fluorescence (a.u.)/mg of protein/min) when compared to vehicle (1.21 ± 0.08 Δ fluorescence (a.u.)/mg of protein/min) (Fig. 10). Rubiscolin-6 didn’t decrease the Caspase-3 activity when compared to the vehicle. A significant decrease in Caspase-3 activity was observed in the presence of all tested analogs. Analog **1** at the dose of 10 µM, which couldn’t decrease the ROS production and the MMP depolarization, showed the potency to suppress the Caspase-3 activity this time. Analog **5** was active only at the highest dose, while analogs **3** and **7** inhibited the stimulation of Caspase-3 activity induced by 6-OHDA most efficiently, suggesting their anti-apoptotic effect.

### Western Blot Analysis

**Fig. 11:**
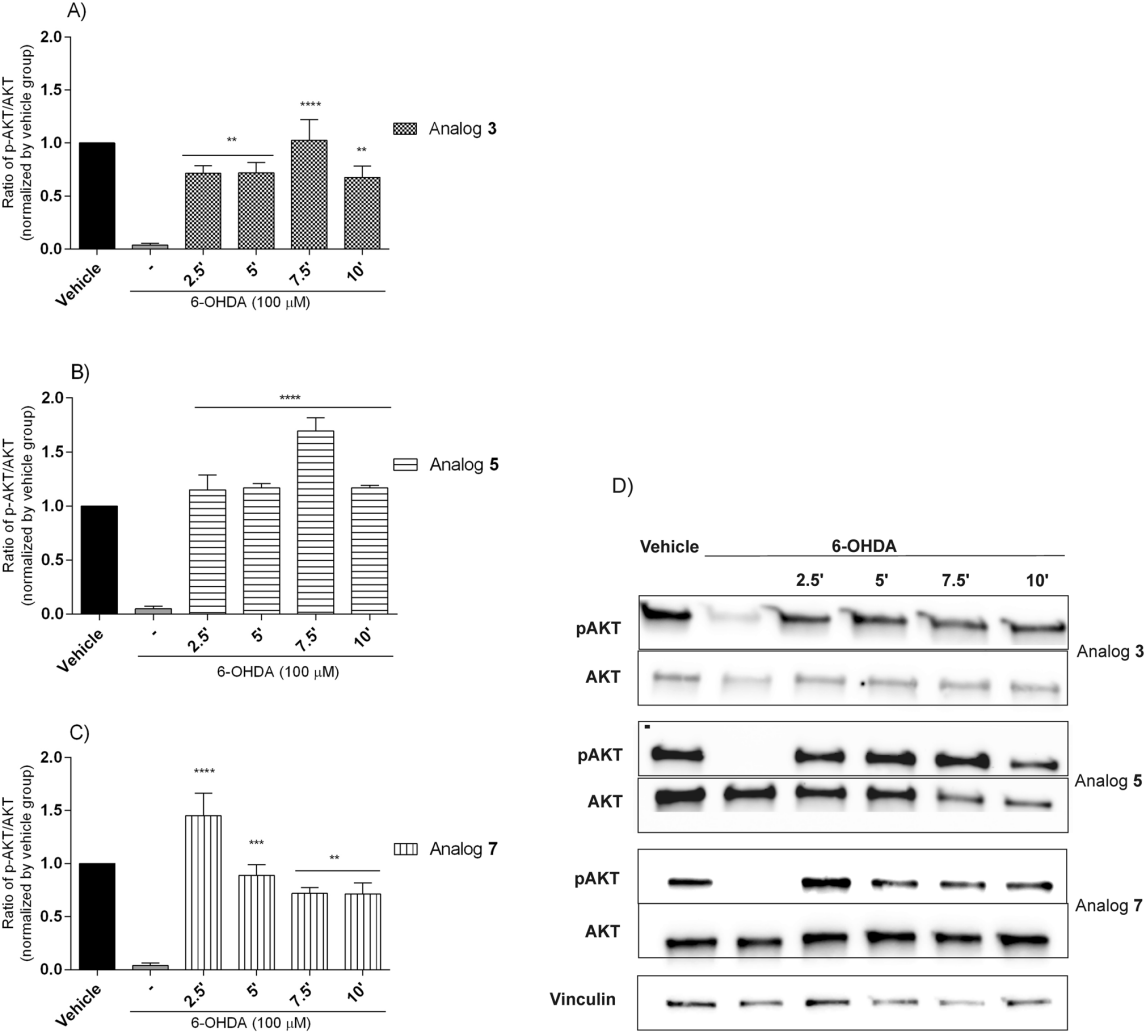
Time-dependent stimulation of AKT phosphorylation by peptides: **A** analog **3**, **B** analog **5** and **C** analog **7** (at the dose of 10 µM) in 6-OHDA-treated SH-SY5Y cells. Cell lysates (20 µg protein) were analyzed for AKT (60 kDa) activation by Western blotting using a phosphor-specific AKT antibody (p-AKT). The same samples were subsequently analyzed on a separate blot using an antibody recognizing unphosphorylated (total) AKT to confirm equal loading on each lane. **D** The phosphorylation level was quantified by densitometry, and the data were normalized to values obtained from vehicle cells. The values in each column represent the mean ± SEM of 3 or 4 independent experiments. Symbols represent significant differences (one–way ANOVA, Dunnett’s test), **p < 0.01, ***p < 0.001, ****p < 0.0001, as compared to 6-OHDA; (−) 6-OHDA

To explore whether increased SH-SY5Y cells’ viability induced by the most potent analogs **3**, **5** and **7** (at the dose of 10 µM) was related to enhanced phosphorylation of AKT at Ser473, cells were incubated with peptides and 6-OHDA, then cell extracts analyzed by Western blotting. Figure 11 showed that three peptides transiently stimulated AKT phosphorylation. The maximum effect was achieved after 7.5 min of stimulation with analogs **3** and **5**, while analog **7** needed only 2.5 min. Incubation with 6-OHDA blocked AKT phosphorylation.

**Fig. 12:**
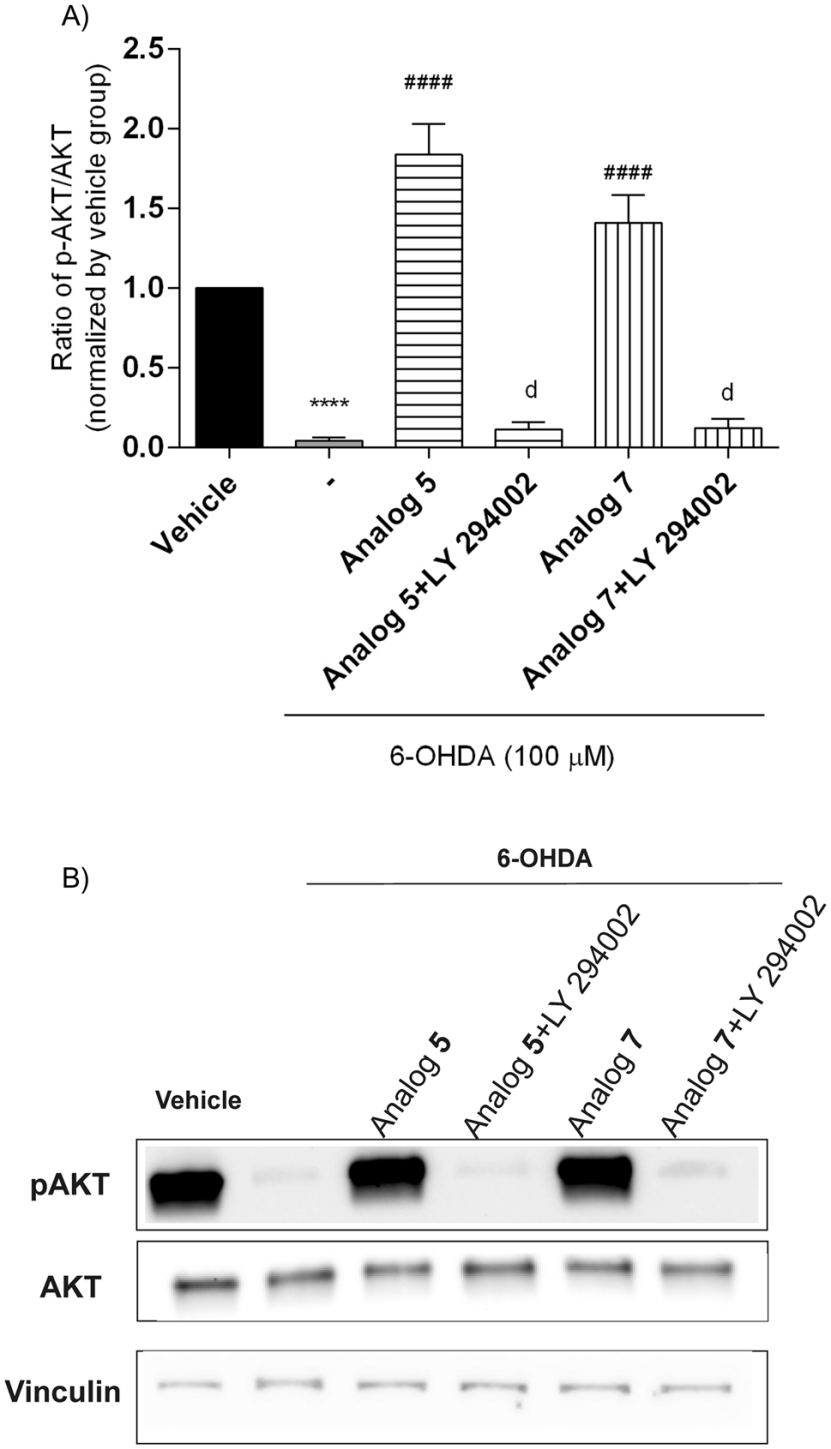
Stimulation of AKT phosphorylation by **A** peptides **5** (incubation time = 7.5 min) and **7** (incubation time = 2.5 min) at the dose of 10 µM in 6-OHDA-treated SH-SY5Y cells. Cell lysates (20 µg protein) were analyzed for AKT (60 kDa) activation by Western blotting using a phosphor-specific AKT antibody (p-AKT). The same samples were subsequently analyzed on a separate blot using an antibody recognizing unphosphorylated (total) AKT to confirm equal loading on each lane. **B** The phosphorylation level was quantified by densitometry, and the data were normalized to values obtained from vehicle cells. The values in each column represent the mean ± SEM of 3 or 4 independent experiments. Symbols represent significant differences (one–way ANOVA, Dunnett’s test), ****p < 0.0001, as compared to the vehicle; ^####^*p* < 0.001 as compared to 6-OHDA; d *p* < 0.0001as compared to analog alone; (−) 6-OHDA. (−) 6-OHDA

We further explored whether peptide stimulation of AKT phosphorylation requires PI3-K incorporation. We incubated SH-SY5Y cells with LY 294002 (PI3-K inhibitor, 20 µM) for 30 min before peptides (10 µM) and 6-OHDA (100 µM) addition. Stimulation was terminated by aspiration of the medium after 7.5 min in the case of analog **5**, and after 2.5 min in the case of analog **7** (according to previous results shown in Fig. 11). In peptides-treated groups, AKT phosphorylation increased compared with 6-OHDA- and LY 294002-treated groups (Fig. 12). Pre-treatment with LY 294002 as PI3-K inhibitor significantly blocked the neuroprotective effects of peptides **5** and **7**. Western blot analysis confirmed that the best peptides in this series prevent neuronal cell death induced by 6-OHDA by modulating the activity of the PI3-K/AKT pathway.

**Fig. 13:**
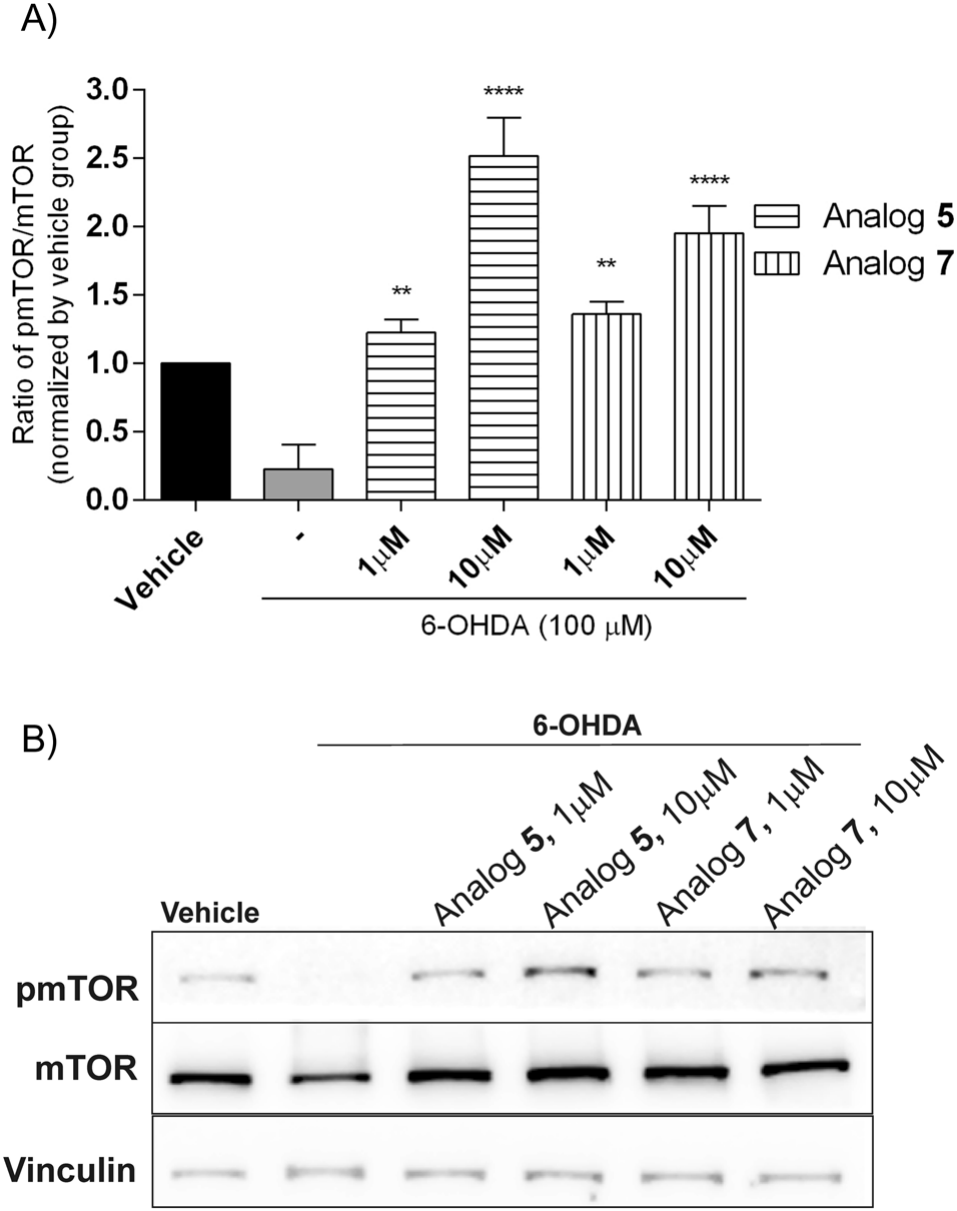
Stimulation of mTOR phosphorylation by **A** peptides **5** and **7** at the dose of 1 and 10 µM in 6-OHDA-treated SH-SY5Y cells. Cell lysates (20 µg protein) were analyzed for mTOR (289 kDa) activation by Western blotting using a phosphor-specific mTOR antibody (p-mTOR). The same samples were subsequently analyzed on a separate blot using an antibody recognizing unphosphorylated (total) mTOR to confirm equal loading on each lane. **B** The phosphorylation level was quantified by densitometry, and the data were normalized to values obtained from vehicle cells. The values in each column represent the mean ± SEM of 3 independent experiments. Symbols represent significant differences (one–way ANOVA, Dunnett’s test), **p < 0.01, ****p < 0.0001, as compared to 6-OHDA. (−) 6-OHDA

The mammalian target of rapamycin (mTOR) signaling is also an important serine/threonine protein kinase in the downstream pathway of PI3-K/AKT. It can promote cell growth, differentiation, survival and inhibit apoptosis signals [34]. As shown in Fig.13, treatment with analogs **5** and **7** (at the dose of 1 and 10 µM, 24h) induced a significant increase of phosphorylated mTOR compared with the 6-OHDA-injured group. The result showed that the PI3-K/AKT/mTOR pathway was activated after treating SH-SY5Y cells with peptides with the highest neuroprotective potency.

### The Effect of Peptides on Inflammatory Response

The in vitro anti-inflammatory effect of R-6 and its analogs on LPS-induced RAW 246.7 macrophages was studied. To directly compare the biological response, we used sub-toxic concentrations of the peptides pre-defined on RAW 254.7 cells; therefore, the R-6 and analogs **1**–**5** and **7** at concentrations equal to 0.1, 1 and 10 µM were used. No cytotoxic activity against RAW 264.7 cells was observed after incubating all studied peptides (Supplementary data, Fig. S17).

**Fig. 14:**
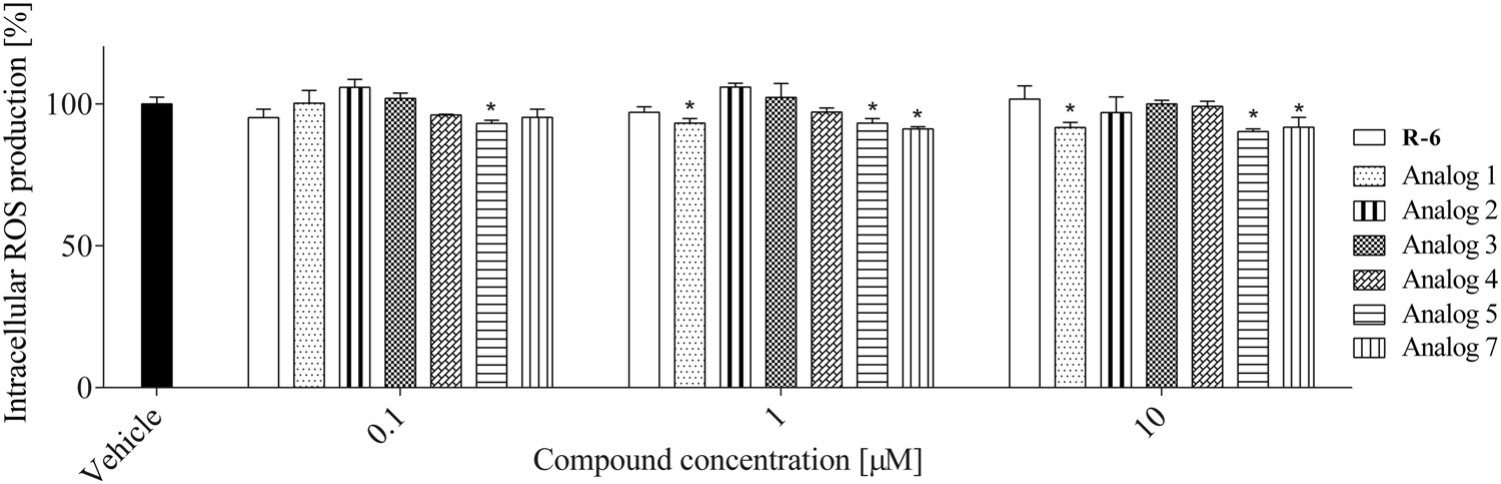
The effect of pre-treatment with peptides (0.1–10 µM) on intracellular ROS generation in RAW 264.7 cells determined with DCFH‐DA assay upon 24 h of incubation. The values in each column represent the mean ± SEM of three or four independent experiments. Symbols represent significant differences (ANOVA, Dunnett’s test), *p < 0.05 as compared to vehicle

Since ROS are involved in cellular signaling and inflammatory response induction, the levels of ROS were determined on RAW 264.7 cells when treated with peptides. As shown in Fig. 14, pre‐incubation of cells with R-6 and analogs** 2**–**4** did not affect intracellular ROS levels. On the other hand, analogs** 1**, **5** and **7** reduced ROS levels by 10–15% at concentrations 1 and 10 µM.

**Fig. 15:**
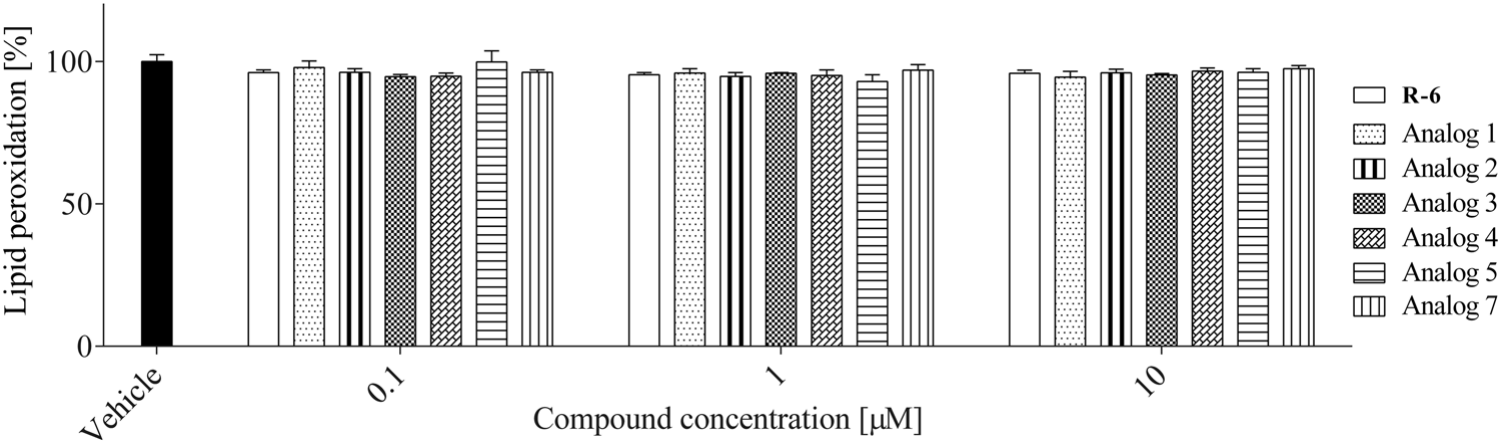
The effect of peptides (0.1–10 µM) on lipid peroxidation in RAW 264.7 cells determined with DPPP probe upon 24 h of incubation. Control cells were only exposed to the vehicle. The values in each column represent the mean ± SEM of three or four independent experiments, and statistical significance was calculated against the vehicle

Since the excessive accumulation of ROS leads to oxidative stress and damage lipids, the peptides’ influence on lipids peroxidation was determined with DPPP probe (Fig. 15), which incorporates into the membranes. The peptides at studied concentrations did not affect lipids peroxidation of macrophage cell membranes cultured under normal conditions.

#### Nitric Oxide (NO) Production in LPS-Stimulated RAW 264.7 Cells

**Fig. 16:**
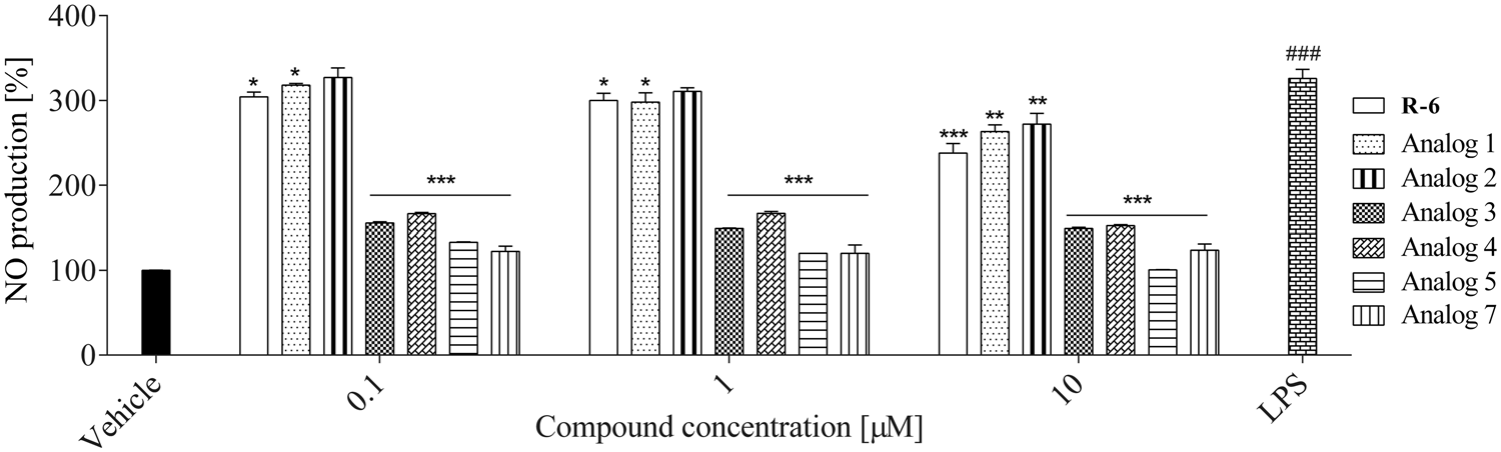
The effect of peptides (0.1–10 µM) on nitric oxide (NO) production in LPS-stimulated RAW 264.7 cells determined with Griess reagent upon 24 h of incubation. Control cells were only exposed to the vehicle. The values in each column represent the mean ± SEM, of three or four independent experiments. Symbols represent significant differences (ANOVA, Dunnett’s test), *p < 0.05, **p < 0.01, ***p < 0.001 when compared to LPS-treated cells and ^###^p < 0.001 when compared to vehicle

Nitric oxide (NO), which is synthesized by nitric oxide synthase (NOS) in activated macrophages, is the primary inflammatory mediator. Since chronic inflammation is related to neurodegenerative diseases, many studies have evaluated the effects of anti-inflammatory agents on the direct inhibition of NO production [26–38]. Nitric oxide levels in LPS-stimulated RAW 264.7 cells were quantified in the presence or absence of peptides (Fig. 16). The exposure of RAW 264.7 to external bacterial toxins, like LPS, has been extensively described to stimulate the secretion of NO, which is produced by the inducible isoforms of nitric oxide synthase (iNOS). As presented in Fig. 16, cells treatment with LPS significantly increased the NO levels, which exceeded 330% compared to the unstimulated control. Tested peptides showed inhibitory effects on LPS-induced NO production; at the dose of 10 µM all the peptides protected macrophages against releasing this mediator. The lowest impact was observed for R-6 and its **1 – 2** analogs, which decreased the NO release by 40–70% compared to LPS-treated cells. The most significant NO reduction was mediated by compound **5** (10 µM), which was generated in a manner comparable to the level determined in unstimulated cells. Considering the lowest concentration (0.1 µM), the analogs **3**, **4**, **5**, and **7** most effectively reduced NO production. Among the studied peptides, the most effective was analog** 7**, which was able to reduce NO level to almost 125%. But still, the increase of these analogs’ concentration from 0.1 to 10 µM was not followed with the proportional inhibition of NO release, demonstrating that 0.1 µM concentration with the anti-inflammatory sufficient effect.

#### Intracellular ROS Formation in LPS-Stimulated RAW 264.7 Cells

**Fig. 17:**
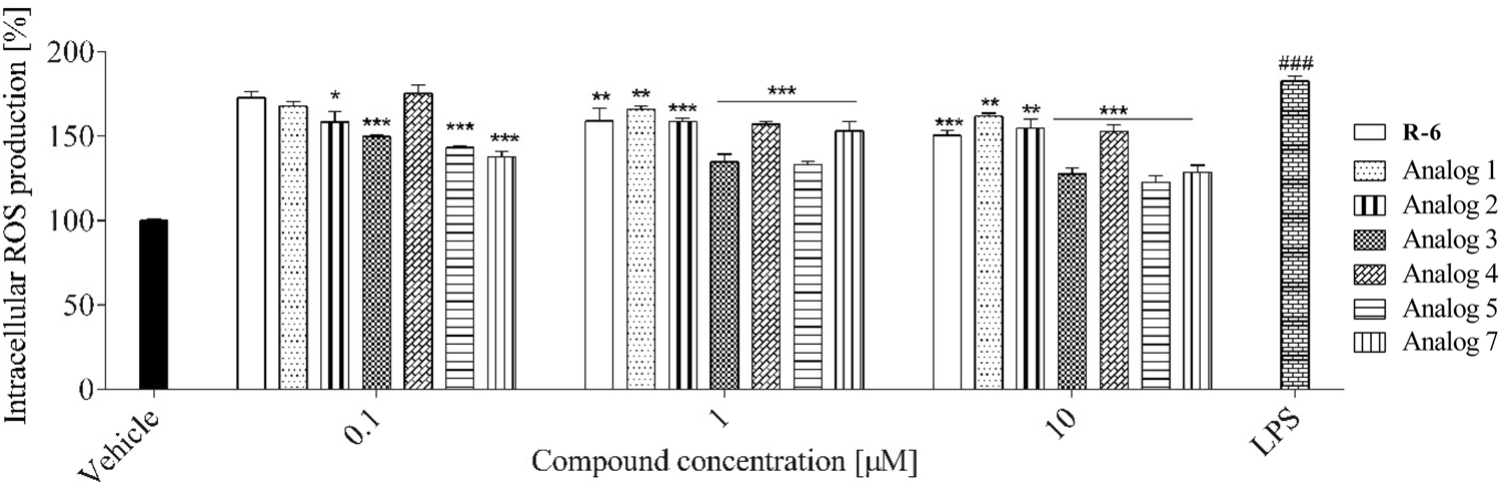
The effect of peptides (0.1–10 µM) on intracellular ROS generation in LPS-stimulated RAW 264.7 determined with DCFH‐DA assay upon 24 h of incubation. Control cells were only exposed to the vehicle. The values in each column represent the mean ± SEM of three or four independent experiments. Symbols represent significant differences (ANOVA, Dunnett’s test), *p < 0.05, **p < 0.01, ***p < 0.001 when compared to LPS-treated cells and ^###^*p* < 0.001 when compared to vehicle

Enhanced production of NO inflammatory marker, which belongs to endogenous free radical species, correlates with oxidative stress generation. Thus, the intracellular level of ROS was studied to determine the potential anti-oxidative properties of peptides. As shown in Fig. 17, in LPS-stimulated RAW 264.7 cells, the ROS level increased almost twice compared to the control. After incubation with studied compounds, levels of ROS decreased by 20–70%. The most effective at the 0.1 µM concentration was analog** 7,** reducing ROS levels by about 50%, but analogs** 3** and **5** also revealed a protective effect. On the other hand, R-6, along with analogs **1–2** and **4**, diminished ROS production with an efficiency not exceeding 40%.

**Fig. 18:**
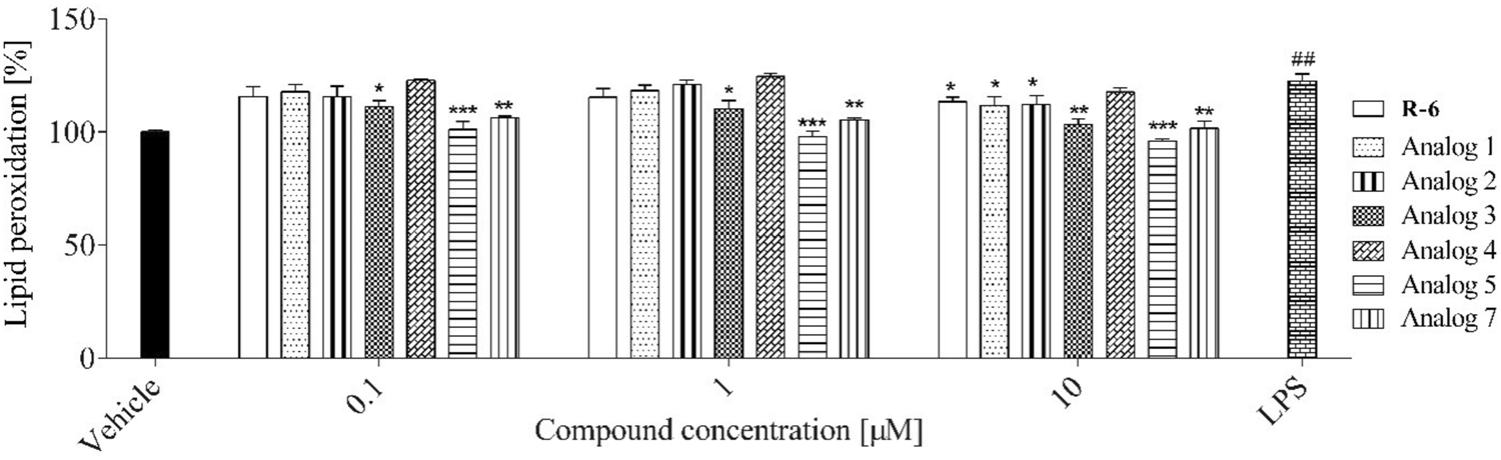
The effect of peptides (0.1–10 µM) on lipid peroxidation in LPS-stimulated RAW 264.7 cells determined with DPPP probe upon 24 h of incubation. Control cells were only exposed to the vehicle. The values in each column represent the mean ± SEM of three or four independent experiments. Symbols represent significant differences (ANOVA, Dunnett’s test), *p < 0.05, **p < 0.01, ***p < 0.001 when compared to LPS-treated cells and ^##^*p* < 0.01 when compared to vehicle

Further analysis revealed that analogs **3**, **5** and **7** could also decrease membrane lipids’ oxidation by almost 10% at the lowest concentration used (Fig. 18). The most significant inhibition of peroxidation was observed for analog **5** at the dose of 0.1 µM. Still, the effect was not better despite higher doses. Parent peptide, R-6 revealed no effect.

### Locomotor Activity

**Fig. 19:**
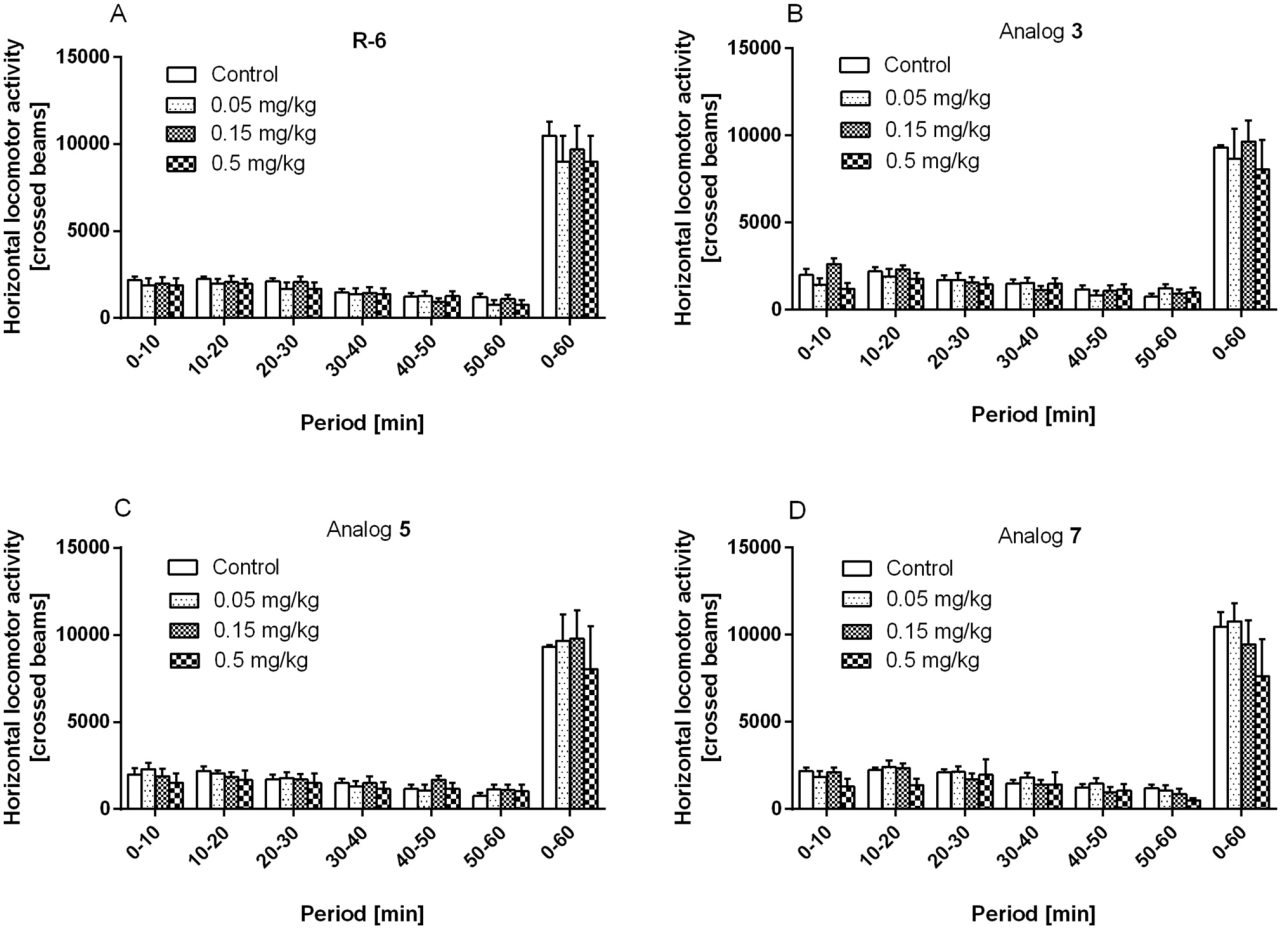
Effect of R-6 (**A**), analog **3** (**B**), analog **5** (**C**), analog **7** (**D**) on horizontal locomotor activity. Mice were injected intracerebraventricular (icv) with vehicle (control) or peptide and placed in the actimeters 10 min after the injection. Horizontal displacements were measured for six consecutive periods of 10 min. Data represent the mean ± SEM of 8–10 mice per group

To be sure that the neuroprotective- and anti-inflammatory- activity of R-6 and its analogs **3**, **5** and **7** do not reduce or increase motor activity, the spontaneous locomotor activity (LMA) test was performed using healthy mice. The influence of peptides was measured over six consecutive 10 min periods (Fig. 19A–D). The spontaneous locomotor activity test showed that tested peptides do not affect locomotor activity. The analysis of total distance moved revealed similar motor responses of the groups across treatment phases. The results obtained were comparable without any significant difference between them.

## Discussion

Rubiscolin-6, as a naturally occurring, linear peptide isolated from the pepsin digestion of D-ribulose-1,5-bisphosphate carboxylase/oxygenase (RuBisCo) [5, 6], has been shown to possess some interesting activities. It exhibited antinociceptive, orexigenic, anxiolytic-like, and memory-enhancing activities in mice after oral administration [5, 8–11]. Additionally, it activates opioid receptors to enhance glucose uptake in skeletal muscle [13] and exerts an antidepressant-like effect in restraint-stressed mice [14]. Our earlier study [17] showed that R-6 produced a potent neuroprotective effect in in vitro model of PD. Our findings suggest that this plant-derived peptide exerts protective effects, possibly related to an anti-oxidation mechanism and an ability to increase BDNF. In the present study, we described the neuroprotective and anti-inflammatory potency of R-6 analogs in which a five-membered pyrrolidine ring of Pro was substituted by a six-membered piperidine ring, with a carboxyl group in position 3 or 4 (*(R)*Nip and Inp, respectively). Additionally, we proposed the introduction of Dmt instead of Tyr^1^, and the amidation of the C-terminus. All proposed changes are well known in opioid peptide science, especially the Dmt substitution [39, 40], and have been successfully explored in our previous studies [21–25]. Generally, earlier opioid peptide structure–activity studies revealed important alterations in the activities through receptor affinity elevation and selectivity and bioactive profile modification [21–25, 40]. In this study, changes proposed turned out to be crucial for three analogs: **3** (Tyr-Inp-Leu-Asp-Leu-Phe-OH), **5** (Dmt-Inp-Leu-Asp-Leu-Phe-OH) and **7** (Tyr-Inp-Leu-Asp-Leu-Phe-NH_2_), probably because of the proper orientation of amino acid in position 1 and 3, which are believed to play an essential role in the biological properties of modified peptides. Generally, the receptor binding and functional activity tests revealed that R-6 analogs display a relatively weak affinity for opioid receptors and potency to stimulate these receptors. Only R-6 and analog **6** (Tyr-Pro-Leu-Asp-Leu-Phe-NH_2_) showed antioxidant potential to reduce iron ions in FRAP assay and displayed the scavenging capacity in ORAC assay. Treatment with analogs **3**, **5** and **7** protects SH-SY5Y cells from toxicity induced by 6-OHDA administration by increasing cell viability, reducing intracellular ROS production and MMP depolarization, and inhibiting Caspase-3 activity.

Phosphoinositide 3-kinase (PI3-K) and its downstream AKT are activated by a wide variety of stimuli (insulin, growth factors, cytokines and cellular stresses) and regulate diverse biological processes such as growth, proliferation and cellular survival [41]. Our MTT results showed that PI3-K/AKT, but not MAPK, modulates the neuroprotective effect of tested peptides because the protective effect was abolished when cells were pre-treated with LY 294002 (PI3-K inhibitor). Western blot analysis confirmed that selected analogs transiently stimulated AKT phosphorylation. Maximum effect was observed after 2.5 (analog **7**) or 7.5 (analogs **3** and **5**) minutes of stimulation, while 6-OHDA completely attenuated AKT phosphorylation. Pre-incubation with LY 294002 prevented peptides-induced AKT phosphorylation, which confirmed that the peptide’s protective effect was likely mediated via the PI3-K/AKT pathway. Additionally, we observed the phosphorylation of mTOR after incubation with peptides; thus, the results proved that PI3-K/AKT/mTOR signal is involved in the neuroprotective mechanism.

Synthesized peptides decreased the NO secretion (analogs **5** and **7**) and intracellular ROS formation (analogs **3**, **5** and **7**) in LPS-stimulated RAW 264.7 cells, which confirmed their anti-inflammatory activity. Neuroinflammation is a widely accepted contributor to the pathogenesis of neurodegenerative diseases because excessive or out-of-control activation of an innate and adaptive immune response in the central nervous system (CNS) is frequently associated with neuronal damage [42–44].

Till now, several studies have explored the mechanism of neuroprotective and anti-inflammatory actions of natural or synthetic compounds. Obtained findings may have important translational implications for treating PD patients in the future. Ghosh et al. [45] evaluated the anti-inflammatory, antioxidant and neuroprotective efficacy of diapocynin, an oxidative metabolite of the naturally occurring agent apocynin. Orally administrated diapocynin (300 mg/kg/day, mice) significantly protected against 1-methyl-4-phenyl-1,2,3,6-tetrahydropyridine (MPTP)-induced striatal dopamine depletion, glial activation in the substantia nigra (SN), iNOS expression, activation of microglial NADPH oxidase. Moreover, diapocynin inhibits the formation of nitrotyrosine and hydroxynonenal in the nigral dopaminergic neurons, improves locomotor activities in MPTP-toxicity and halts the disease progression in a chronic MPTP model.

Neuroprotective effect in PD animal model through inflammation pathway has been proven for Optimised Yinxieling Formula (OYF), a Chinese medicine consisting of *Curcuma zedoaria*, *Sarcandra glabra*, dark plum fruit, Rhizoma Smilacis Glabrae, *Lithospermum erythrorhizon*, *Paeonia lactiflora*, and *Glycyrrhiza uralensis*, well-known formula exerts therapeutical effect and antiinflammation property on psoriasis [46]. The studies showed that OYF exerts anti-inflammatory effects, which might be related to the protection of dopaminergic neurons in 6-OHDA-induced chronic neurotoxicity. In the PD animal model, OYF reversed the motor behavioural dysfunction, upregulated the tyrosine hydroxylase (TH) expression, decreased the immunoreactivity of ionized calcium-binding adapter molecule 1 (Iba-1) and glial fibrillary acidic protein (GFAP), and downregulated the mRNA levels of TNF-α and COX-2. In in vitro study, OYF significantly decreased the mRNA levels of TNF-α, IL-1β, IL-6, iNOS, and COX-2.

The well-known neuroprotective peptide humanin (HN) showed an anti-inflammatory effect because it inhibits astrocyte overactivation induced by lipopolysaccharide. Additionally, pre-treatment with HN decreased the level of proinflammatory cytokines, interleukin (IL)-6, IL-1β and tumour necrosis factor α (TNFα) induced by LPS [47].

Additionally, our results indicated that R-6 and analogs **3**, **5**, **7** do not reduce or increase the motor activity of healthy mice in LMA test performed using the locomotor activity. Further experimental in vivo studies are needed to determine the role of R-6 analogs in the 6-OHDA-induced effects on neuronal cells.

## Conclusions

The present study revealed that R-6 analogs with Inp in position 2 possessed neuroprotective and anti-inflammatory activity that could be valuable in preventing or treating PD. These peptides prevented neuronal death via attenuation of ROS, mitochondrial dysfunction and Caspase-3 activity. Selected peptides alleviate 6-OHDA injury by activating the PI3-K/AKT/mTOR signaling pathway. They were able to ameliorate LPS-mediated inflammation in macrophages via inhibition of intracellular generation of ROS and NO production. However, future studies need to characterize precisely the underlying mechanisms of injury and death that lead to neurodegeneration and explain which mediators functionally impair cognition, motion or memory.

### Supplementary Information

Below is the link to the electronic supplementary material.

Supplementary file1 (DOCX 1082 KB)

## Data Availability

The authors declare that the data supporting the findings of this study are available within the paper and its Supplementary Data files. Should any raw data files be needed in another format they are available from the corresponding author upon reasonable request.
