# Supplementary material for: Neuroprotective and Anti-inflammatory Effects of Rubiscolin-6 Analogs with Proline Surrogates in Position 2
Source: Neurochem Res. 2023 Dec 20;49(4):895–918. doi: 10.1007/s11064-023-04070-z (PMC10901950; doi:10.1007/s11064-023-04070-z)
Supplement: Supplementary file 1 — Supplementary file1 (DOCX 1082 KB) [file 11064_2023_4070_MOESM1_ESM.docx]

**Supplementary data**

**Neuroprotective and anti-inflammatory effects of rubiscolin-6 analogs with proline surrogates in position 2.**

Renata Perlikowska^1*^, Joana Silva^2^, Celso Alves^3^, Patricia Susano^2^, Małgorzata Zakłos-Szyda^4^, Agnieszka Skibska^1^, Anna Adamska-Bartłomiejczyk^1^, Karol Wtorek^1^, Jean-Claude do Rego^5^, Jean-Luc do Rego^5^, Alicja Kluczyk^6^, Rui Pedrosa^3^

*^1^ Department of Biomolecular Chemistry, Faculty of Medicine, Medical University, Lodz, 92-215, Poland*

*^2^* *MARE—Marine and Environmental Sciences Centre, ARNET - Aquatic Research Network, Politécnico de Leiria, 2520-630 Peniche, Portugal*

*^3^* *MARE—Marine and Environmental Sciences Centre, ARNET - Aquatic Research Network, ESTM, Politécnico de Leiria, 2520-614 Peniche, Portugal*

*^4^* *Institute* *of Molecular and Industrial Biotechnology, Faculty of Biotechnology and Food Sciences, Lodz University of Technology, Stefanowskiego 2/22, 90-537 Lodz*

*^5^ University of Rouen Normandy, Platform of Behavioural Analysis (SCAC), Inserm US51 - CNRS UAR2026 HeRaCLes, Institute For Reseach and Innovation in Biomedicine (IRIB), Rouen, France*

*^6^ Faculty of Chemistry, University of Wroclaw, 50-383 Wroclaw, Poland*

**Contents:**

Physicochemical characterization of rubiscolin-6 (R-6) and its analogs **1-7** (Table S1)………………………………………………………………………. p. 2

High resolution MS spectra (Fig. S1-S8) …………………………......... p. 3-10

^1^H NMR spectra (700 MHz, DMSO‑d_6_) of R-6 and analogs 1–7 (Fig. S9-S16) …… p.11-18

The effects of peptides (0.1 – 10 µM) on RAW 264.7 cells' metabolic activity

determined with MTT assay upon 24 h of incubation (Fig. S17)………. p. 19

The original immunoblots………………………………………………. P.20-22

**Table S1**. Physicochemical characterization of R-6 and its analogs **1-7**.

| Analog | Sequence | Formula | Formula weight  g/mol | m/z  calculated  [M+H]^+^ | m/z  found  [M+H]^+^ |
| --- | --- | --- | --- | --- | --- |
| R-6 | H-Tyr-Pro-Leu-Asp-Leu-Phe-OH | C_39_H_54_N_6_O_10_ | 766.8803 | 767.3974 | 767.3993 |
| 1 | H-Dmt-Pro-Leu-Asp-Leu-Phe-OH | C_41_H_58_N_6_O_10_ | 794.9334 | 795.4287 | 795.4260 |
| 2 | H-Tyr-(*R*)-Nip-Leu-Asp-Leu-Phe-OH | C_40_H_56_N_6_O_10_ | 780.9068 | 781.4131 | 781.4111 |
| 3 | H-Tyr-Inp-Leu-Asp-Leu-Phe-OH | C_40_H_56_N_6_O_10_ | 780.9068 | 781.4131 | 781.4096 |
| 4 | H-Dmt-(*R*)-Nip-Leu-Asp-Leu-Phe-OH | C_42_H_60_N_6_O_10_ | 808.9600 | 809.4444 | 809.4424 |
| 5 | H-Dmt-Inp-Leu-Asp-Leu-Phe-OH | C_42_H_60_N_6_O_10_ | 808.9600 | 809.4444 | 809.4426 |
| 6 | H-Tyr-Pro-Leu-Asp-Leu-Phe-NH_2_ | C_39_H_55_N_7_O_9_ | 765.8955 | 766.4134 | 766.4059 |
| 7 | H-Tyr-Inp-Leu-Asp-Leu-Phe-NH_2_ | C_40_H_57_N_7_O_9_ | 779.9221 | 780.4290 | 780.4192 |

HR-ESI-MS experiments were performed on a Bruker micrOTOF-Q (time-of-flight) mass spectrometer (peptide R-6) an FTICR (Fourier transform ion cyclotron resonance) Apex-Qe Ultra 7 T mass spectrometer (peptides 1-7, Bruker Daltonics, Bremen, Germany) equipped with standard ESI source. The instrument was operated in the positive-ion mode and calibrated with the Tunemix™ mixture (Agilent Technologies, Palo Alto, CA, USA). Peptide solutions (water:acetonitrile 50:50 with 0.1% HCOOH) were introduced at a flow rate of 3 μl/min.

High resolution MS spectra of rubiscolin-6 (R-6) and its analogs **1-7**. In insets, fragment of the experimental spectrum (top panel) is compared with the simulated isotopic profile calculated for the expected molecular formula of protonated species (bottom panel).


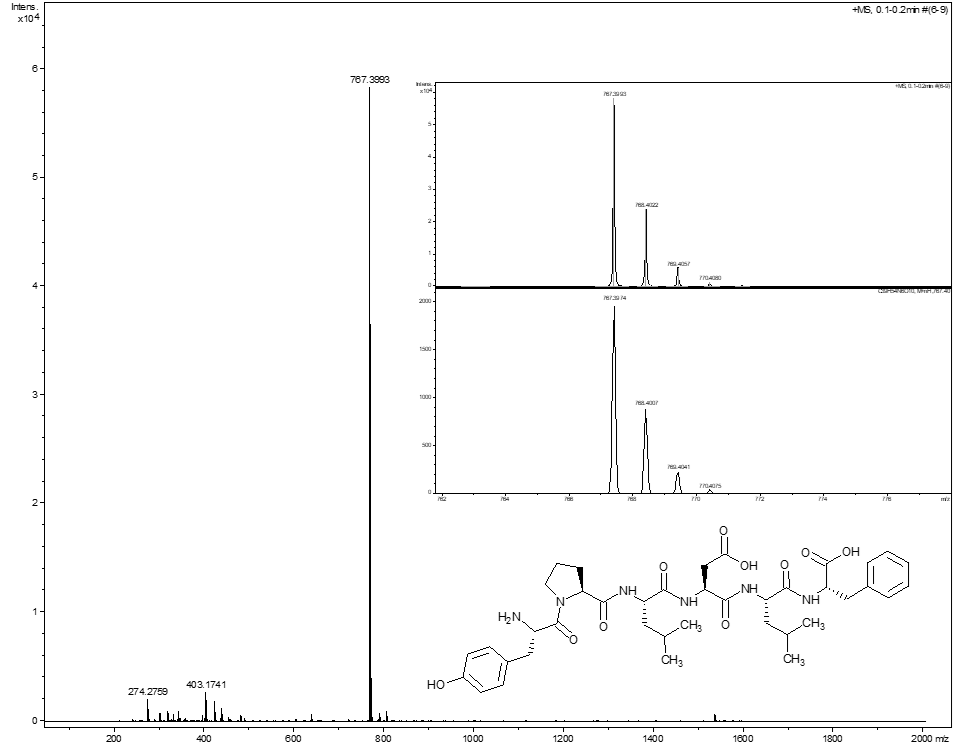


**Fig. S1.** High resolution MS spectrum of rubiscolin-6 (R-6)**.**

**Fig. S2.** High resolution MS spectrum of analog **1.**

**Fig. S3.** High resolution MS spectrum of analog **2**.

**Fig. S4**. High resolution MS spectrum of analog **3**.

**Fig. S5**. High resolution MS spectrum of analog **4**.

**Fig. S6.** High resolution MS spectrum of analog **5**.

**Fig. S7**. High resolution MS spectrum of analog **6**.

**Fig. S8.** High resolution MS spectrum of analog **7**.

**Fig. S9**. ^1^H NMR (700 MHz, DMSO-d_6_) for R-6.

**Fig. S10**. ^1^H NMR (700 MHz, DMSO-d_6_) for Analog **1**.

**Fig. S11**. ^1^H NMR (700 MHz, DMSO-d_6_) for Analog **2**.

**Fig. S12**. ^1^H NMR (700 MHz, DMSO-d_6_) for Analog **3**.

**Fig. S13**. ^1^H NMR (700 MHz, DMSO-d_6_) for Analog **4**.

**Fig. S14**. ^1^H NMR (700 MHz, DMSO-d_6_) for Analog **5**.

**Fig. S15**. ^1^H NMR (700 MHz, DMSO-d_6_) for Analog **6**.

**Fig. S16**. ^1^H NMR (700 MHz, DMSO-d_6_) for Analog **7**.


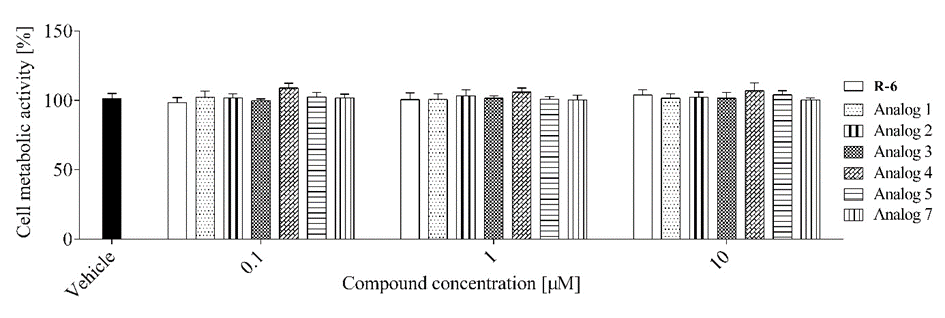


**Fig. S17.** The effects of peptides (0.1 – 10 µM) on RAW 264.7 cells' metabolic activity determined with MTT assay upon 24 h of incubation. Control cells were only exposed to the vehicle. The values in each column represent the mean ± SEM of four independent experiments, and statistical significance was calculated against the vehicle.

The original immunoblots. Protein levels were normalized to Vinculin (cropped bands are shown in red frame) based on densitometry analysis, and relative protein levels are shown.

Corresponding to **Fig. 11D**

**Analog 3**


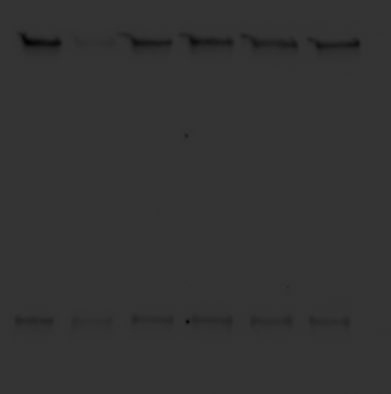


AKT-60 kDa

pAKT-60 kDa

**Analog 5**


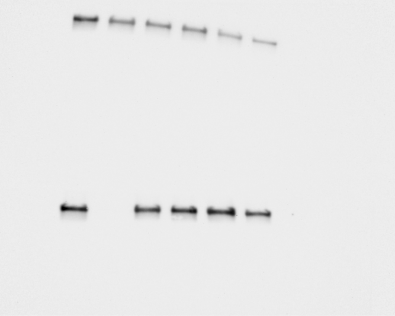


pAKT-60 kDa

AKT-60 kDa

**Analog 7**


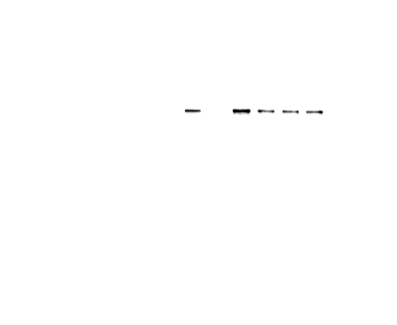


pAKT-60 kDa


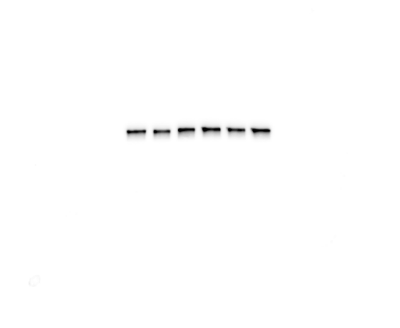


AKT-60 KDa


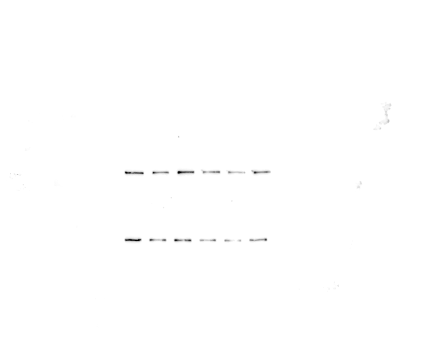


Vinculin-124 kDa

Vinculin-124 kDa

Corresponding to **Fig. 12B**

AKT-60 kDa


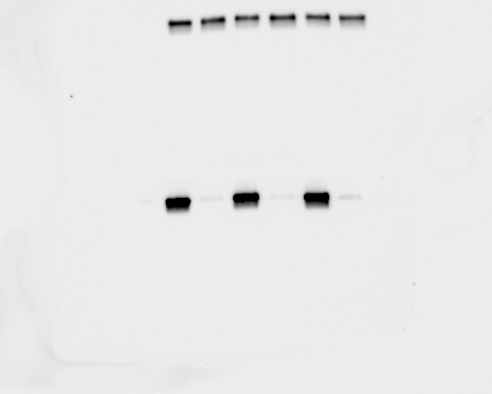


pAKT-60 kDa


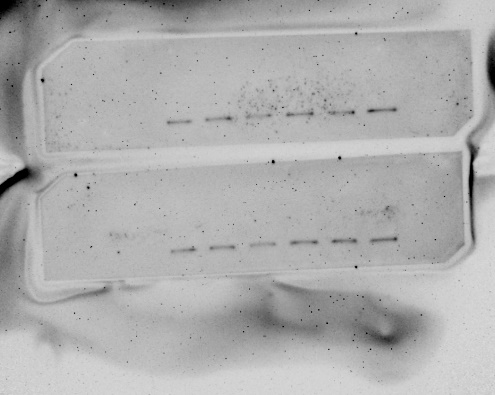


Vinculin-124 kDa

Vinculin-124 kDa

Corresponding to **Fig. 13B**


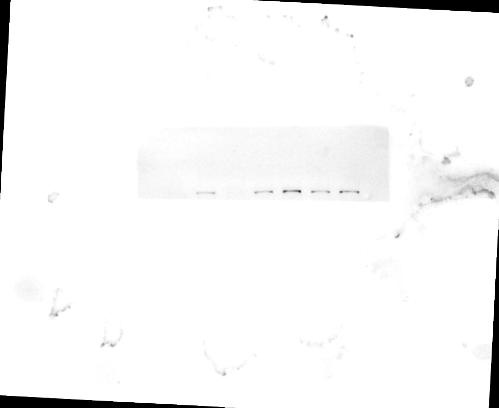


pmTOR-289 kDa


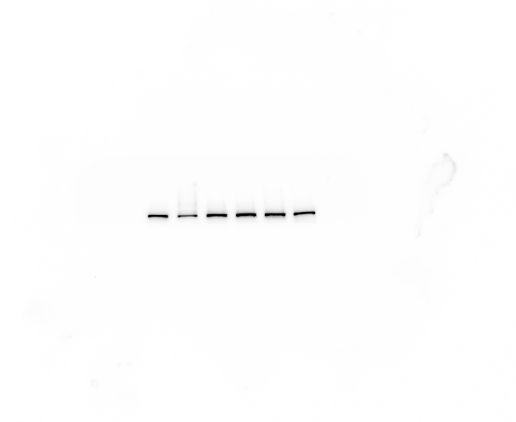


mTOR-289 kDa


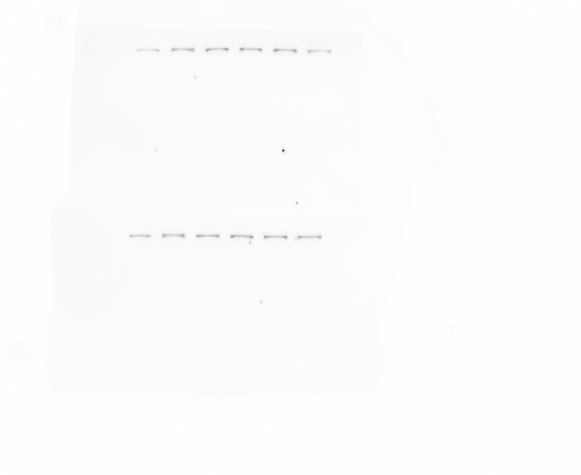


Vinculin-124 kDa

Vinculin-124 kDa
